# A tissue engineering approach to regenerate the cranial suture skeletal stem cell niche with a multicompartment biomaterial scaffold

**DOI:** 10.1038/s41413-026-00539-z

**Published:** 2026-05-28

**Authors:** W. Benton Swanson, Lindsey Douglas, Seth M. Woodbury, Jackson Albright, Haichun Pan, Maiko Omi-Sugihara, Miranda Eberle, Jake Herremans, Hwa Kyung Nam, Rafael Correia Cavalcante, Coral Chen, Peter X. Ma, Nan E. Hatch, Yuji Mishina

**Affiliations:** 1 Department of Biologic and Materials Science, School of Dentistry, University of Michigan, Ann Arbor, MI USA; 2 Department of Chemistry, College of Literature, Science and the Arts, University of Michigan, Ann Arbor, MI USA; 3 Department of Physics, College of Literature, Science and the Arts, University of Michigan, Ann Arbor, MI USA; 4 Department of Biomedical Engineering, College of Engineering, University of Michigan, Ann Arbor, MI USA; 5 Department of Orthodontics and Pediatric Dentistry, School of Dentistry, University of Michigan, Ann Arbor, MI USA; 6 Department of Materials Science and Engineering, College of Engineering, University of Michigan, Ann Arbor, MI USA; 7 Macromolecular Science and Engineering Center, College of Engineering, University of Michigan, Ann Arbor, MI USA; 8 Present Address: Department of Oral Medicine, Infection and Immunity, School of Dental Medicine, Harvard University, Boston, MA USA

**Keywords:** Bone, Pathogenesis

## Abstract

Craniosynostosis is a debilitating congenital anomaly characterized by the premature fusion of cranial sutures in the skull, resulting from the abnormal fate specification of critical skeletal stem cells. This leads to disrupted craniofacial development and dysmorphology. As an alternative to invasive cranial vault remodeling surgery, we propose a tissue engineering approach to effectively restore the damaged or absent suture stem cell niche using a biomaterial capable of regioselective stem cell maintenance, marking a significant paradigm shift. By harnessing the role of biomaterial scaffold pore design to direct cell fate, we developed a “bone-suture-bone” design within a triphasic scaffold. We demonstrate that this unique scaffold design maintains stemness in a central region while promoting osteogenic differentiation in the surrounding areas, replicating the interfaces and function of the native suture. The scaffold’s ability to reconstitute an engineered skeletal stem cell niche and facilitate the functional recovery of the craniosynostosis phenotype is validated in a caBmpr1a; Wnt1-Cre murine model of midline craniosynostosis, the most common nonsyndromic clinical presentation in humans. This work shows significant promise for improving patient outcomes through a rational tissue engineering strategy for reconstituting a native stem cell niche.

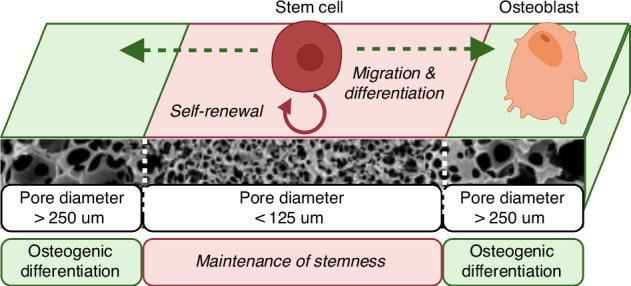

## Introduction

Craniosynostosis is a congenital condition characterized by the premature fusion of one or more cranial sutures in the skull, affecting approximately 1 in 2 500 live births.^1^ The sutures are critical for accommodating rapid brain growth during childhood; their premature fusion leads to increased intracranial pressure, abnormal skull shape, and potential developmental and neurocognitive delays.^2,3^ Traditional treatments to manage craniosynostosis are primarily surgical, namely surgical osteotomy of the fused suture and invasive cranial vault remodeling. Given the lack of replacement with healthy suture tissue in these treatment modalities, re-synostosis (re-fusion of the bones) occurs commonly, requiring additional revision surgeries.^4^ Cranial vault remodeling procedures have become more common due to reduced re-synostosis, although they pose a significantly greater risk for blood loss and adverse events than suturectomy.^5^ A significant unmet clinical need exists, given that surgical complication rates are as high as 22.9%,^6^ and no pharmacologic treatments are available due to the diverse etiology of the disease.^7^ Given the time-limited nature of skull vault growth, transient or temporal growth support is an alternative therapeutic approach, and a scaffold-based tissue-engineering approach is a viable option to restore craniofacial morphology.

Skeletal stem/progenitor cell (SSPC) niches are critical to coordinating the growth and maintenance of skeletal organs.^8^ The cranial suture has been identified as a critical stem cell niche, harboring the *Gli1*^+^,^9^ *Axin2*^+^,^10,11^ and *Prx1*^+12^ populations, with similar yet unique properties to SSPCs identified in long bones,^13–18^ and vertebrae,^19^ attributed to their separate developmental origins. Suture SSPCs are directly involved in bone growth from the suture based on their incorporation into calvarial bone.^9,20^ Rather than a disorder of accelerated bone growth, premature suture fusion is attributed to a loss of the cranial suture stem cells by aberrant differentiation^9,21,22^ or apoptosis.^23,24^ For example, a striking craniofacial phenotype in Gli1-Cre^ERT2^; R26DTA mice results from the ablation of Gli1^+^ SSPCs, resulting in the fusion of all sutures.^9^ In another example, augmented bone morphogenic protein (BMP) signaling in cranial neural crest cells (*Wnt1Cre*; *caBmpr1a*) results in aberrant chondrogenic differentiation and endochondral ossification of the suture, leading to midline craniosynostosis.^22,24,25^

The suture SSPC niche provides anatomical and functional cues to stem cells, coordinating their differentiation in a temporal and spatial context. Thus, the extracellular matrix environment is crucial in maintaining their stemness,^26^ justifying a tissue engineering approach to treating craniosynostosis. Previous attempts to regenerate a suture-like tissue have focused on preventing bone formation rather than addressing the stem cell etiology of the disease.^27,28^ The transplantation and maintenance of an autonomous skeletal stem cell niche, replicating the cellular character of the cranial suture, has yet to be reported. Our group has extensively studied the cranial suture niche^22–24,29–32^ and systematically elucidated key biomaterial properties that regulate biomaterial-directed cell fate specification in vitro and in vivo.^30,33–37^ We demonstrated that scaffold macropore size is a critical parameter that modulates vascularization and the ability to maintain the stemness in heterogeneous stem cell populations from the suture and bone marrow. A sufficiently small pore size (spherical, <125 μm diameter) is required to maintain stemness by providing a mechanoprotective environment to suppress YAP/TAZ signaling. Sufficiently large pores (spherical, >250 μm diameter) promote a robust osteogenic phenotype. We propose a tissue engineering approach to treating craniosynostosis by re-establishing the SSPC niche using a biomaterial construct as an alternative to multiple invasive surgeries. This challenge involves restoring the complex microenvironment of the stem cell niche and surrounding tissue to mimic the endogenous function faithfully.^38–40^

Here, we report the newly developed triphasic scaffold, mimicking the “bone-suture-bone” interface by patterned macropore size. In vitro and in vivo evidence suggests that this design enables the simultaneous maintenance of stemness, facilitated by a region with 60–125 μm diameter pores, and osteogenic differentiation in the flanking areas with 250–425 μm pore diameter. Differentiating, but not naïve, stem cells migrate from the center to osteogenic flanking regions, contributing to the scaffold’s ability to spatially restrict tissue phenotype in vivo. In vivo, the triphasic scaffold is superior to a monophasic osteogenic scaffold in maintaining SSPCs, assessed in a lineage tracing model, and capable of resisting bone formation when challenged by augmented BMP signaling. Critically, we demonstrate the functional recovery of craniofacial dysmorphology in a *caBmpr1a; Wnt1-Cre* model of craniosynostosis resulting from triphasic scaffold implantation at the site of suture fusion. This study offers a foundational demonstration of engineering a transplanted SSPC niche to facilitate the normalized growth and development of skeletal tissues where essential cell populations are lost or altered due to disease processes, representing a translational therapeutic approach. Because craniofacial morphology becomes increasingly constrained after this critical early postnatal craniofacial growth period, we evaluate therapeutic efficacy within a defined window spanning active craniofacial growth, during which transient restoration of a suture-like microenvironment is expected to provide durable phenotypic benefit. In mice, this corresponds to the early postnatal, peri-weaning period (approximately postnatal day 21), which precedes sexual maturation and coincides with rapid craniofacial growth.

## Results

### Engineering a multicompartment scaffold to re-establish a functional skeletal stem cell niche

**Fig. 1:**
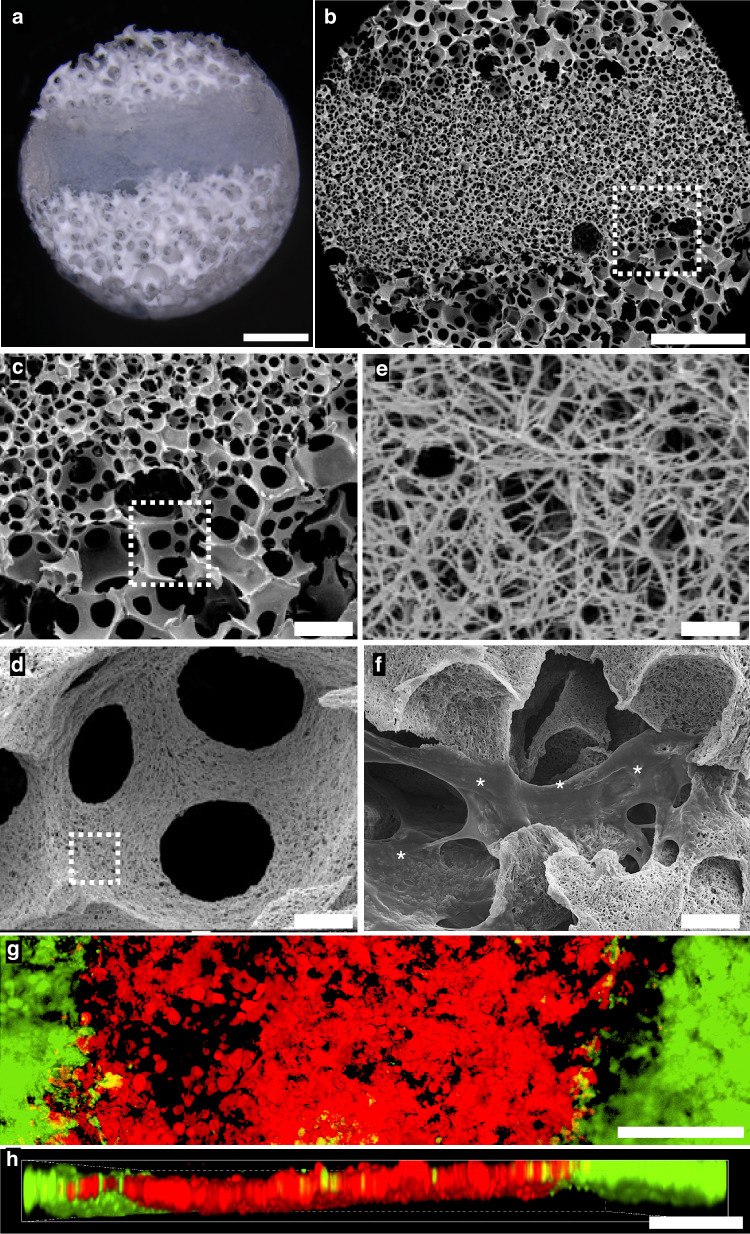
The three distinct regions of the scaffold are well-demarcated, visually (**a**, scale = 1 mm) and by scanning electron micrographs, where a small pore region (center) is flanked by two large pore regions (**b**, scale = 1 mm). The interface between the small and large pore regions is uniformly interconnected and continuous (**c**, scale = 300 μm), and interconnections between macropores allow cell migration and mass transfer (**d**, scale = 50 μm). Dashed lines mark the region of interest in each subsequent panel. Across the scaffold, a nanofibrous surface texture facilitates cell adhesion and protein adsorption (**e**, scale = 1 μm; **f**, scale = 10 μm, cells are marked by asterisks). Fluorescently labeled cells are used to demonstrate regioselective cell seeding (**g**, scale = 0.5 mm) and penetration through the depth of the scaffold (**h**, scale = 2.0 mm) after 24 h of in vitro culture

We designed a multicompartment scaffold from poly (L-lactic acid), PLLA, with the intent to augment the osteogenic capacity of large pore scaffolds with a local stem cell reservoir embedded *within* the osteogenic scaffold microenvironment (“triphasic”), mimicking the cranial suture. Using sugar spheres, a triphasic scaffold with distinct zonal pore sizes is fabricated using a newly developed layer-by-layer sacrificial porogen method (Fig. 1a). Poly L-lactic acid is a biocompatible, biodegradable polymer that is FDA approved and used in a variety of commercial medical applications.^36,41,42^ A region of sufficiently small 60–125 μm diameter spherical macropores is sandwiched between two regions with spherical macropores sized 250–425 μm, designed to support robust osteogenesis and vascularization. The layer-by-layer method of sugar sphere template fabrication ensures uniform pore size in distinct zones. After leaching sugar spheres, scanning electron microscopy shows the scaffold is highly porous, with distinct regions of small and large pores (Fig. 1b). The boundary between the large and small pore regions is well-defined and straight (Fig. 1b, c). The width of the center small pore region is tunable to various width based on the amount of sugar added in this step. Uniform spherical macropores are observed to be interconnected to each other and across their interface, allowing for cell migration throughout the construct and the diffusion of waste and nutrients (Fig. 1d). The scaffold surface is uniformly nanofibrous, with fiber diameters ranging from 50 to 500 nm, attributed to thermally induced phase separation of PLLA at −80 °C^43^ (Fig. 1e). Nanofibers facilitate cell adhesion when cultured with primary cells (Fig. 1f). The triphasic scaffold enables for regioselective cell seeding in different compartments. As a proof of concept, we labeled BMSCs with either green or red membrane fluorescent dye and seeded them to the small pore (red) or large pore (green) region of the scaffold using surgical loupes for magnification (Fig. 1g). After 24 h, the scaffold thickness is well cellularized, demonstrating the cells ability to adhere and penetrate the synthetic matrix (Fig. 1h).

### Spatial boundaries uniquely regulate tissue phenotype in the triphasic scaffold

**Fig. 2:**
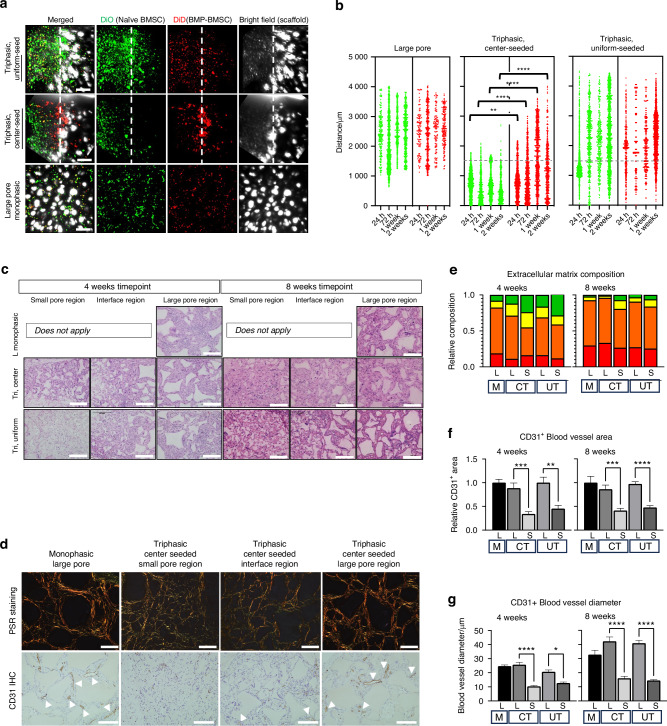
Differential migration is responsible for the maintenance of stemness in the triphasic scaffold. Differentiating BMSCs (red, DiD) and naïve BMSCs (green, DiO) are simultaneously seeded from a mixture to triphasic scaffolds uniformly (top) in the center region only (middle) or to large pore monophasic scaffolds (bottom) and cultured for 24 h, 72 h,1 week and 2 weeks (representative images at one week shown in **a**, scale = 500 μm). The position of the fluorescently labeled cells is quantified and plotted as a function of location (**b**, dashed line demarcates the boundary between the small (below) and large pore (above) regions). Subcutaneous explants are subjected to histologic analysis by hematoxylin and eosin staining (**c**, *n* = 5 per group, scale = 500 μm), picrosirus red (**d**, top, *n* = 5 per group, scale = 500 μm), and CD31 immunohistochemistry (**d**, bottom, *n* = 5 per group, scale = 500 μm). Tissue composition is quantitatively assessed by image analysis of picrosirus red staining to determine extracellular matrix composition (**e**, *n* > 4 images per sample, *n* = 5 biologic replicates per group, colors inside of each bar represent the contributing colors to the picrosirus red stain: green, orange, yellow, red, whose ratio is used to measure tissue maturity and matrix composition). Vascularization is quantitatively assessed by CD31^+^ area image analysis (**f**, *n* > 4 images per sample, *n* = 5 biologic replicates per group; blood vessels are identified by arrowheads, scale = 200 μm) and by CD31^+^ blood vessel diameter (**g**, *n* > 4 images per sample, *n* = 5 biologic replicates per group) at both 4 and 8 weeks. *P < 0.05, **P < 0.01, ***P < 0.001, ****P < 0.000 1. Statistical analysis was carried out in GraphPad Prism v10. Student’s *t* test and two-way ANOVA were used to determine the statistical significance of observed values between experimental groups, where *P* < 0.05 was considered significant. Tukey’s test was used as a single-step method to determine differences between group means, compare multiple means, and contrast statistical significance between groups

MSCs differentiate spontaneously in vivo and in vitro^44^ and must be able to mobilize to contribute to tissue growth and repair.^45,46^ Therefore, rather than prohibiting osteogenic differentiation with a region of stem cell maintenance only, the flanking large pore regions provide an osteogenic environment for the subsets of cells migrated out from the central region to mimic the bone-suture-bone configuration and thus aim to balance between maintenance of stem cells and their differentiation, compared to a monophasic osteogenic scaffold (monophasic large pore). We first aimed to characterize the behavior of naïve versus differentiating stem cells in the scaffold. We subjected primary BMSCs from mice to osteogenic differentiation by administration of recombinant bone morphogenic protein (rhBMP2, 50 ng/mL) (Fig. S1A). When each cell type is seeded to the center of the triphasic scaffold, naïve MSCs remained in the center while differentiating mineralizing, BMP-treated BMSCs (BMP-BMSC) migrated to the flanking region (Fig. S1A). BMP-BMSC were combined in a 1:1 ratio with naïve, freshly isolated BMSCs and seeded together either in the center region of triphasic scaffolds, uniformly on triphasic scaffolds, or uniformly on monophasic scaffolds (control) and cultured for 2 weeks. Confocal microscopy was used to analyze the position and distribution of fluorescently labeled cells over time (Fig. 2a, b). When seeded from the center, we observed that osteogenically committed BMP-BMSC cells migrate at a significantly greater rate and distance toward the flanking large pore region than naïve BMSCs, which do not migrate. Migration of BMP-BMSCs from the center small pore region begins as early as 24 h and maintains a steady rate between 72 h and 2 weeks. Conversely, a 1:1 mixture of BMP-treated and naïve BMSCs in large pore or triphasic scaffolds shows little self-organization and remains heterogeneously distributed.

To confirm our hypothesis about differential migration in vivo, we seeded cells to scaffolds similarly and subcutaneously implanted the cell-scaffold constructs in wild-type adult mice. Subcutaneous explants prepared for histologic observation demonstrated that all scaffolds were well cellularized after four weeks of in vivo culture (Fig. 2c). Picrosirus red staining reflects spatially modulating extracellular matrix composition phenotype by pore size (Fig. 2d, Fig. S1B). At both 4 and 8 weeks, there is a uniform distribution of total ECM throughout the constructs, while there is a greater abundance of green (immature matrix) in the small pore region of triphasic constructs compared to the large pore region of constructs or monophasic large pore control (Fig. 2e, Table S1). The colors green, yellow, orange, and red represent relative maturity of the collagen matrix. Green represents thin, immature collagen fibers, while yellow, orange, and red represent progressively thicker and more mature collagen fibers. Red and orange are the thickest, most mature. Quantitative CD31^+^ immunohistochemistry (Fig. 2d) indicates that the small pore region attenuates vascular ingrowth and maturation, restricted to the center region, at 4 and 8 weeks (Fig. 2f, Fig. S1C). Large pore regions of the triphasic scaffold facilitated robust vascularization, like monophasic control, demonstrating no negative effect of the small pore region on the osteogenic potential of the large pore regions (Fig. 2f, g). Finally, we observed that the small pore region facilitates the upregulation of SSPC stemness markers (Fig. S1D), congruent with the maintenance of an immature and less vascularized matrix. Interestingly, triphasic scaffolds seeded from the center showed a superior ability to modulate the maintenance of stemness. The large pore region of triphasic scaffolds, subjected to either seeding protocol, upregulates markers of osteogenesis similarly to monophasic large pore scaffolds.

### Lineage tracing the fate of stemness in the triphasic scaffold

Next, we sought to determine the in vivo maintenance of stemness using a putative SSPC lineage-tracing model and determine these cells’ contribution to bone formation. *HoxA11* is a critical gene in skeletal progenitor/stem cell function that gives rise to all skeletal lineages throughout life in mice and humans.^47^ *HoxA11* expressing SSPCs differentiate into *LepR*^+^ and *Osx*^+^ cells^47,48^ and can be marked by knocking in eGFP to the *HoxA11* locus.^48^ Unlike other SSPC markers not specific to the stemness process, despite marking SSPC populations (e.g., *Gli1*), *HoxA11* provides a *specific* measure of skeletal stem cells, endorsing its suitability to determine the ability of the triphasic scaffold to modulate maintenance of stemness spatially. Cultured in vitro, bone marrow stem cells from *HoxA11eGFP* mice (Fig. S2A) lose GFP signal in large (250–425 μm) pore scaffolds after only 1 week of in vitro culture and nearly attenuated after two weeks (Fig. S2B). On the other hand, small (60–125 μm) pore monophasic scaffolds maintain GFP expression and maintenance of *HoxA11*-expression for up to three weeks in vitro. To confirm the functionality of HoxA11eGFP as a measure of stemness, we demonstrate that osteogenic differentiation induced by osteogenic medium or osteogenic medium supplemented with recombinant BMP2 (50 ng/mL) reduces the expression of GFP (Fig. S2C).

**Fig. 3:**
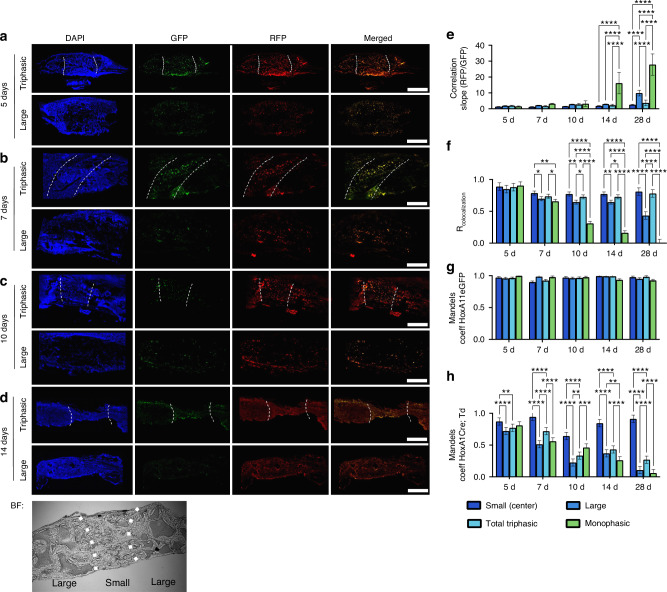
Lineage tracing the fate of SSPC in the triphasic scaffold. Lineage tracing using cells isolated from HoxA11Cre+; TdTomato/+; HoxA11eGFP+ mice. The RFP signal indicates progeny of HoxA11^+^ skeletal stem cells, and the GFP signal indicates expression of HoxA11 at the time of observation. The yellow signal indicates RFP (TdTomato) and GFP expression, indicating stemness maintenance. Cell-scaffold constructs in calvarial defects are explanted after 5 days (**a**, scale = 1 mm), seven days (**b**, scale = 1 mm), 10 days (**c**, scale = 1 mm), and 14 days (**d**, scale = 1 mm) and assessed histologically. White dashed lines demarcate the boundary between small (S) and large (L) pore regions in triphasic scaffolds, which is determined from the bright-field channel of each image, shown representative at bottom. The correlation between the RFP and GFP signals is computed using the Costes threshold correlation analysis method. The slope of the linear regression fit is plotted in (**e**), and Pearson’s correlation coefficient is in (**f**). The Mander’s split colocalization coefficients for the GFP signal (**g**) and RFP signal (**h**) are derived at each time point. All data are plotted as mean ± standard deviation. * *P* < 0.05, ** *P* < 0.01, ***P < 0.001, ****P < 0.000 1. Data represent a minimum sample size of *n* > 4. Statistical analysis was carried out in GraphPad Prism v10. Student’s *t* test and two-way ANOVA were used to determine the statistical significance of observed values between experimental groups, where *P* < 0.05 was considered significant. Tukey’s test was used as a single-step method to determine differences between group means, compare multiple means, and contrast statistical significance between groups

To trace the fate of skeletal stem cells in our triphasic scaffold in vivo, we isolated BMSCs from tamoxifen-induced *HoxA11Cre*^*ERT2*^; *TdTomato*; *HoxA11eGFP* mice (Fig. S2D). Cells initially expressing *HoxA11* show red fluorescence from Td-tomato; cells that maintained their stemness also show green fluorescence from the *HoxA11eGFP* allele (Fig. S2E). Primary cells from tamoxifen-induced *HoxA11Cre*^*ERT2*^, *TdTomato, and HoxA11eGFP* mice were seeded onto triphasic or monophasic scaffolds and then implanted in parietal bone defects in vivo. Cell-scaffold constructs were explanted from calvariae at various time points up to 28 days and prepared for histologic analysis by confocal laser microscopy (Fig. 3a-d). We analyzed the colocalization of GFP and RFP signals using the Costes method as a readout of cells to maintain stemness and progenies of the SCCs.^49^ Over time, the large pore region of the triphasic scaffold and large pore monophasic scaffold significantly skew towards RFP expression, as indicated by the increased correlation slope (Fig. 3e). The expression of RFP and GFP remain well-correlated in the triphasic scaffold by Pearson’s correlation coefficient, while the correlation in large pore monophasic scaffolds diminishes by 28 days (Fig. 3f). The degree of colocalization is further assessed by Mander’s method,^50^ where zero indicates no colocalization, and 1.00 indicates perfect colocalization. In the case of GFP expression, the Manders coefficient remains above 0.90. It is the greatest in the small pore region of the triphasic scaffold (Fig. 3g), indicating near-perfect colocalization of the RFP signal with the GFP signal. On the other hand, the colocalization of the GFP signal when the RFP signal is present (i.e., maintenance of stemness) significantly depends on the region within the triphasic scaffold (Fig. 3h).

### The triphasic scaffold resists bone formation with locally augmented BMP signaling

**Fig. 4:**
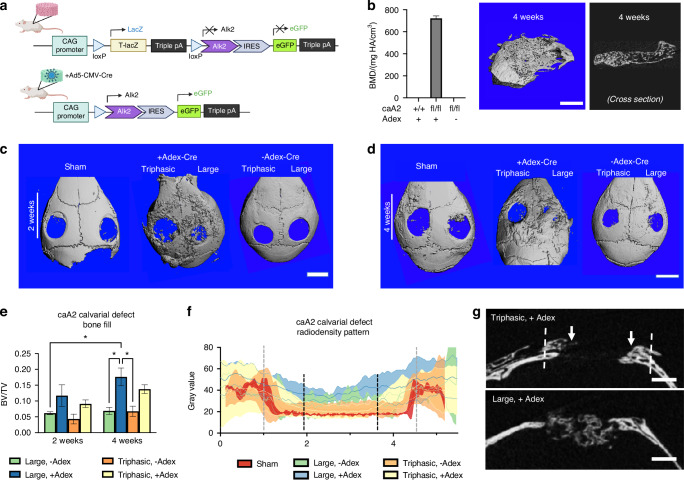
The triphasic scaffold resists aberrant osteogenic differentiation in the central region. Adenoviral Cre (Adex-Cre) is used to locally induce the ALK2Q207D mutation (caA2) as shown schematically in (**a**). Large pore PLLA scaffolds loaded with Adex-Cre were implanted subcutaneously in mice; after 4 weeks, scaffolds were explanted and assessed for bone formation (**b**, *n* = 4, *p* < 0.0001 for all comparisons; scale = 1 mm). Adex-Cre-laden triphasic (left side, *n* = 6) and large pore (right side, *n* = 6) scaffolds were implanted in a bilateral 3.5-mm calvarial defect model in caA2 mice, then assessed after two (**c**, scale = 3.5 mm) and 4 weeks (**d**, scale = 3.5 mm). Bone fill (**e**, *n* = 6 samples per group) and spatial distribution of bone fill within the defect site (**f**; gray dashed line = defect boundary; black dashed line = L-S boundary within triphasic scaffold; each plot represents the average of *n* = 6 samples per group and *n* = 3 sections per sample) is measured by micro CT, with representative sections shown in **g** (*n* = 6 per group). Bone radiodensity is plotted across the coronal plane, bisecting the midpoint of the defect to assess differences in neotissue distribution. All data are plotted as mean ± standard deviation. *P < 0.05, **P < 0.01, ***P < 0.001, ****P < 0.000 1. Statistical analysis was carried out in GraphPad Prism v10. Student’s *t* test and two-way ANOVA were used to determine the statistical significance of observed values between experimental groups, where *P* < 0.05 was considered significant. Tukey’s test was used as a single-step method to determine differences between group means, compare multiple means, and contrast statistical significance between groups

The ideal tissue-engineered suture stem cell niche must maintain the stemness of skeletal stem cells in the engineered suture and simultaneously prevent bone formation in the niche. Yet, the tissue engineering construct must integrate within the surgical site such that the engineered bone and endogenous bone heal to form a contiguous tissue, enabling cell migration from the niche toward the bone compartments. To assess the triphasic scaffold’s ability to resist bone formation, we used mice carrying a Cre-inducible transgene encoding the BMP receptor, ALK2^Q207D^ (a human gene with Q207D mutation, caALK2^fl^), which is a constitutively active mutant.^32^ Cre-mediated recombination leads to the expression of ALK2^Q207D^ driven by the CAG promoter (Fig. 4a), resulting in excess BMP signaling. Local recombination of Cre is achieved by local administration of the viral vector Ad5-CMV-cre (Adex-Cre), which we previously reported to induce heterotopic ossification following intramuscular injection.^51^ To establish the use of Adex-Cre to cause local recombination in scaffolds, we subcutaneously implanted large pore monopḍhasic scaffolds laden with Adex-Cre in *UbiCreER*^*+*^ and *TdTomato*^*+*^ mice. Histologically, the RFP signal matched systemic tamoxifen treatment, showing effective local recombination. The fluorescent signal from these cells is maintained within the scaffold post-explantation after 4 weeks. (Fig. S3A). To validate subcutaneous ectopic bone formation, we bred mice to generate compound homozygous mice for ALK2Q207D and mTmG. Micro-CT and histology show recombination (Fig. S3B) and local ectopic bone formation in the scaffold (Fig. 4b), with no distal systemic bone phenotype (Fig. S3C, D).

We sought to test the hypothesis that the small pore region of the triphasic scaffold is sufficient to resist bone formation even in the presence of augmented osteogenic signaling. Adex-cre-loaded scaffolds were implanted in bilateral parietal bone defects in adult caALK2^fl/fl^ mice, compared to PBS control (no virus). Bone formation was observed after 2 and 4 weeks (Fig. 4c, d). Compared to the triphasic scaffold, the large pore scaffold facilitates greater new bone tissue formation at both time points when caALK2 is recombined based on bone volume fraction within the defect site (Fig. 4e). The triphasic scaffold promotes new bone formation, although less than the large pore monophasic scaffold and spatially restricted, resisting defect in-fill in the center region. Radiopacity was measured in CT scans through the center of the engineered tissue, perpendicular to the long axis of the skull (bisecting the L-S-L interface) (Fig. 4f, representative sections shown in Fig. 4g), demonstrating that sham defects do not significantly heal (Fig. 4f; gray dashed line indicates defect margin), while robust bone fill is observed in large monophasic scaffolds with Adex-Cre. In contrast, bone formation is observed but spatially restricted to the outer thirds in the case of triphasic scaffolds with Adex-Cre (black dashed lines indicate triphasic scaffold L-S interface). The constructs are well-cellularized after 2 weeks, indicating that the pores do not inhibit infiltration as a Supplementary figure (Fig. S3E).

### Surgical suturectomy and engineered stem cell niche implantation in vivo

Our group and others have reported the craniosynostosis phenotypes of transgenic mice with gain-of-function and loss-of-function mutations for BMP signaling within cranial neural crest cell populations, mimicking human patients.^11,22,24^ Mice expressing a constitutively active form of *Bmpr1a* (*caBmpr1a*), encoding BMP type 1A receptor in a neural crest-specific manner (*Wnt1-Cre*), develop short snouts, round faces, hypertelorism, and metopic suture fusion.^22^ *Bmpr1a* plays a crucial role and has been identified as essential for skeletal stem cell (SSPC) self-renewal and SSPC-mediated bone formation,^11^ justifying our choice of this model.

**Fig. 5:**
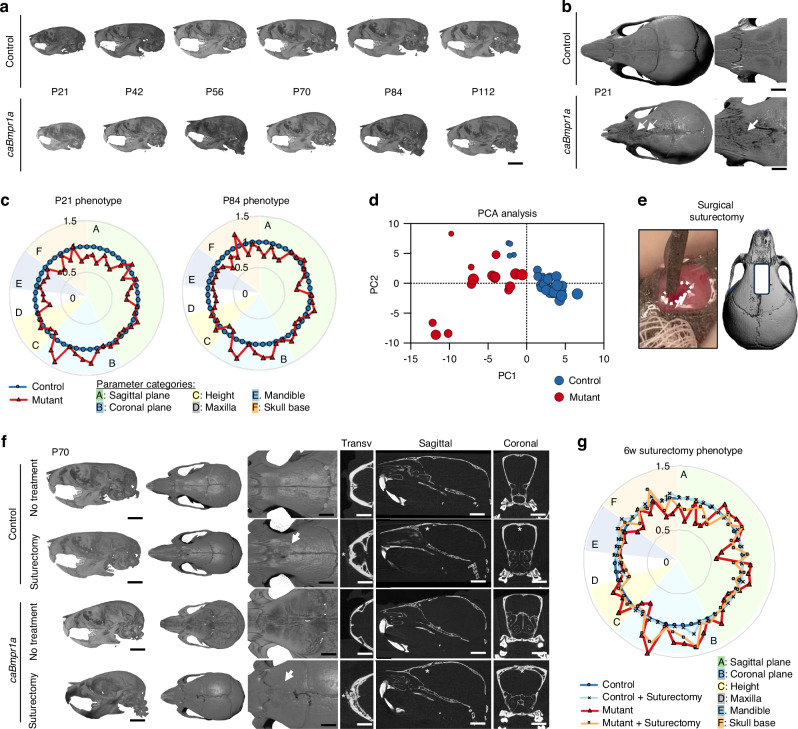
caBmpr1a; Wnt1-Cre mutant mice demonstrate a robust craniosynostosis phenotype and re-synostosis in the case of surgery. caBmpr1a; Wnt1-Cre mutant mice demonstrate a marked craniofacial phenotype throughout life, assessed by microcomputed tomography, compared to Cre-negative mice (control, **a**). At P21, the Wnt1-Cre; caBmpr1a mutant demonstrates the premature fusion of the internasal and metopic cranial sutures (arrow, **b**), compared to control mice, where the metopic suture remains patent throughout life. Disease burden is measured as a function of craniofacial phenotype from morphometric measurements of microcomputed tomography scans, shown as a spider plot at P21 and P84 (**c**). The contribution of each of the six measurement categories overlays the radar plot (from the top, clockwise: sagittal plane, coronal plane, height, maxilla, mandible, skull base). Principle component analysis is used for dimensionality reduction (circle size increases with age, **d** We developed a surgical suturectomy model whereby the fused cranial suture (or equivalent region) is excised using a piezoelectric surgical handpiece (**e**). After 4 weeks, littermate control (no treatment) and surgical suturectomy-treated mice are assessed by microcomputed tomography to determine suture synostosis (**f**; cross sections are shown in the transverse, sagittal, and coronal planes; areas of fusion after surgery are marked by the white arrow and asterisks). Morphometric analysis to determine the effect of the surgical intervention on craniofacial shape is summarized in a spider plot (**g**; blue = control without surgery, light blue = control + suturectomy, red = mutant without surgery, orange = mutant + suturectomy). All data are reported as mean ± standard deviation and represent a minimum sample size of *n* > 4. Statistical analysis was carried out in GraphPad Prism v10. Student’s *t* test and two-way ANOVA were used to determine the statistical significance of observed values between experimental groups, where *P* < 0.05 was considered significant. Tukey’s test was used as a single-step method to determine differences between group means, compare multiple means, and contrast statistical significance between groups. Male and female mice were included in all analyses unless explicitly stated otherwise

Here, we established that this craniofacial phenotype observed in *caBmpr1a* (mutant) mice persists throughout life (e.g., beyond the weaning-stage craniofacial growth period, as previously described) (Fig. 5a). The metopic suture is fused in *caBmpr1a* mutant mice by P21 (Fig. 5b, white arrows). We developed a landmark-based method to measure the morphometric disease manifestation quantitatively (Table 1, Fig. S4) and demonstrated significant discrepancies in skull length, internal angles, width, and skull height in both weaning-stage craniofacial growth period (P21) and adulthood (P80), summarized across 49 features in a spider plot at each time point (Fig. 5c, Table S2A, B). Principal component analysis (PCA) reveals distinct clustering of control and mutant mice (Fig. 5d, Table S2C), where PC1 and PC2 account for 56.85% of the cumulative proportion of variance, providing strong evidence of the impact of the mutation on skull shape in the craniosynostosis mouse model. To assess the effect of a transplanted engineered SSPC niche on disease severity and craniofacial sequelae, we developed a surgical suturectomy procedure using a piezoelectric surgical handpiece and diamond bur to excise the fused metopic suture while respecting adjacent and underlying anatomy (ethmoid bone, nasal sinuses, dura mater) at P42 (Fig. 5e). After 4 weeks, re-synostosis is observed in both suturectomy-treated control and caBmpr1a mutant mice, based on bone fill at the defect site leading to fusion across the parietal bones (Fig. 5f white arrows, Fig. S5A). The suturectomy alone, without scaffold treatment, does not alleviate the craniofacial phenotype of *caBmpr1a* mice (Fig. 5g, Fig. S5B, Table S3).

**Table 1:** List of landmarks used for craniofacial morphometric analysis

Labels (sample ID)	Sample	Cage number and ear punch
	Scan	uCT Scan ID
Categorical Variables	Genotype	C = control, P = P0Cre(+), M = Wnt1-Cre(+)
	Cohort	Intervention Group
	Trial	Month of Surgery
	Sex	M = male, F = female
	Age	Age at time of scan
	Surgery	Y = yes (surgical suturectomy + triphasic scaffold implantation), N = no
	Severe	Y = yes, N = no
Body Weight	BW SX	Body weight at DOS
	BW 4w	Body weight at terminal point
Landmarks	Points	Description
	1	Anterior border of nasal bone
	2	Frontal bone-nasal bone junction, along midline
	3	Parital bone-frontal bone junction, at intersection of sagittal and coronal sutures
	4	Interparietal-parietal bone junction, at intersection of sagittal and lambdoid sutures
	5	Inferior midpoint of interparietal bone
	6	Occipital crest
	7 R	Right midpoint of lateral interface between nasal bone and premaxilla
	7 L	Left midpoint of lateral interface between nasal bone and premaxilla
	8 R	Right anterior maxilla
	8 L	Left anterior maxilla
	9 R	Right lacrymal bone, anterior border
	9 L	Left lacrymal bone, anterior border
	10 R	Interface of right zygomatic bone and maxilla
	10 L	Interface of left zygomatic bone and maxilla
	11 R	Right intersection between coronal, squamosal, and sphenofrontal sutures
	11 L	Left intersection between coronal, squamosal, and sphenofrontal sutures
	13	Inferior boundary of the sphenofrontal suture
	14	Anterior boundary of tympanic bulla
	15	Anterior boundary of the maxilla
	16	Incisive foramen
	17	Anterior boundary of the maxilla
	18	Anterior border of the palate
	19	Posterior border of the palate
	20	Posterior border of the condyle
	21	Posterior, inferior border of the angular process
	22	Anterior, inferior apex of the alveolar process
	23	Inferior border of the incisive canal
	24	Superior border of the incisive canal
	25	Anterior border of preshenoid bone
	26	Anterior border of sphenoid bone
	27	Anterior border of basisphenoid bone
	28	Posterior border of basisphenoid bone
Sagital Plane Measurements (Skull Length)	B (mm)	Total skull length
	A (mm)	Anterior skull length
	P (mm)	Posterior skull length
	A/P	Ratio of anterior to posterior skull length
	L1 (mm)	Nasal bone length
	L2 (mm)	Frontal bone length
	L3 (mm)	Parietal bone length
	L4 (mm)	Interparietal lenth
	L5 (mm)	Occipital length
	L6 (mm)	Total distance along bone surface
	L1/L6	Ratio of L1 to L6
	L1/L2	Ratio of L1 to L2
	A1 (¬∞)	Angle made by B-L1
	A2 (¬∞)	Angle made by L1-L2
	A3 (¬∞)	Angle made by L2-L3
	A4 (¬∞)	Angle made by L3-L4
	A5 (¬∞)	Angle made by L4-L5
	A6 (¬∞)	Angle made by L5-B
Coronal Plane Measurements (Skull Width)	W7 (mm)	Nasal bone width
	W8 (mm)	Facial width
	W9 (mm)	Inter-orbital width
	W10 (mm)	Frontal width
	W11 (mm)	Skull width
	W12 (mm)	Orbital width
	W7/L1	Ratio of nasal width to nasal length
	W11/P	Ratio of skull width to posterior skull length
	W7/W11	Ratio of nasal width to posterior width
Transverse Plane Measurements (Skull Height)	H13 (mm)	Facial height
	H14 (mm)	Anterior cranial height
	H15 (mm)	Posterior cranial height
Maxilla	M16 (mm)	Premaxilla
	M17 (mm)	Maxilla
	M18 (mm)	Palate
Mandible	J19 (mm)	Posterior mandible height
	J20 (mm)	Upper mandible length
	J21 (mm)	Lower mandible length
	J22 (mm)	Anterior mandibular height
Skull Base Length	B1 (mm)	Presphenoid length
	B2 (mm)	Basisphenoid length
	B3 (mm)	Basioccipital length
	B (mm)	Total skull base length
	B1/B2	Ratio of presphenoid to basisphenoid
	B2/B3	Ratio of basisphenoid to basioccipital
	B1/B3	Ratio of presphenoid to basioccipital

**Fig. 6:**
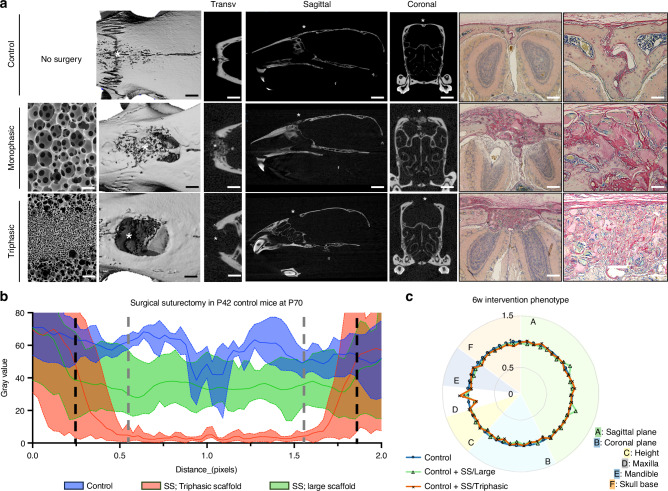
Triphasic scaffold is required for maintenance of a suture-like structure. Large pore monophasic and triphasic scaffolds seeded with BMSCs were implanted in control mice at P42 (6 weeks) and observed after four weeks by microcomputed tomography and histologically by pentachrome (**a**, *n* = 3 per group; asterisks mark key regions of the MicroCT sections). Large pore monophasic scaffolds (middle) facilitated significantly greater bone fill and stochastic neotissue organization than the triphasic scaffold (bottom). Bone radiodensity is plotted across the coronal plane, bisecting the midpoint of the defect to assess differences in neotissue distribution (**b**, *n* = 3 samples per group; 3 sections per sample). No alteration in the phenotype of control mice was observed (**c**). All data are reported as mean ± standard deviation. Statistical analysis was carried out in GraphPad Prism v10. Student’s *t* test and two-way ANOVA were used to determine the statistical significance of observed values between experimental groups, where *P* < 0.05 was considered significant. Tukey’s test was used as a single-step method to determine differences between group means, compare multiple means, and contrast statistical significance between groups. Male and female mice were included in all analyses unless explicitly stated otherwise

Next, we hypothesized that the triphasic scaffold is critical for maintaining the stem cell niche and organizing bone formation within the surgical defect to prevent re-synostosis. To assess this, we performed the surgical suturectomy in control mice at P42 (6 weeks) and implanted either a large pore monophasic (250-425 μm diameter) or triphasic scaffold. The metopic suture of control mice remained patent throughout life, as expected. The large pore scaffold was sufficient to facilitate suture synostosis (fusion); stochastic woven bone formation is observed in the scaffold’s internal structure (Fig. 6a, Fig. S6A). On the other hand, the triphasic scaffold prevented re-synostosis and maintained a patent engineered suture-like tissue. Radiodensity quantification suggests that the triphasic scaffold compartmentalizes new bone formation from the edges of the defect (Fig. 6b, black dashed lines) abutting to but not extending across the boundary of the small and large pore regions of the scaffold (gray dashed lines). In contrast, the large pore scaffold facilitates bone fill throughout the defect. Neither the large nor triphasic scaffold caused appreciable alterations in craniofacial morphology in control mice (Fig. 6c, Fig. S6B, Table S5).

### Surgical suturectomy and triphasic scaffold implantation normalize the burden of craniosynostosis in mice

**Fig. 7:**
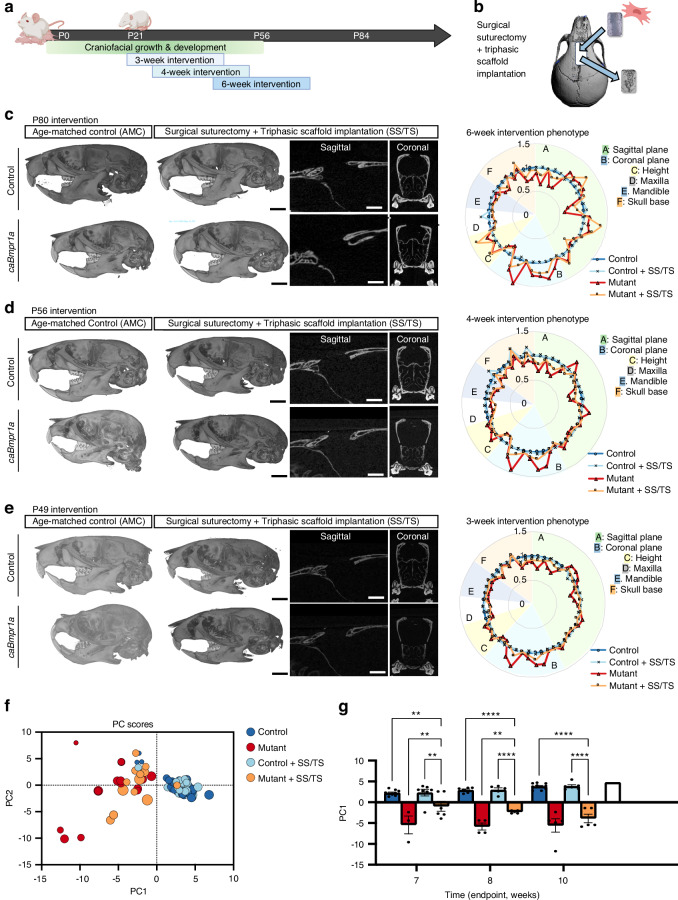
Triphasic scaffold alleviates the disease phenotype of caBmpr1a craniosynostosis. Weaning-stage control and caBmpr1a mice were subjected to treatment at P21 (3 weeks), P28 (4 weeks), and P42 (6 weeks), as shown schematically in (**a**), by surgical suturectomy and triphasic scaffold implantation (**b**). After 4 weeks, age-matched control (AMC) and surgical suturectomy + triphasic scaffold implantation (SS/TS) treated mice are assessed by microcomputed tomography (**c**: P42 intervention at P80; **d**: P28 intervention at P56; **e**: P21 intervention at P49). The effects of the SS intervention on craniofacial shape are summarized in spider plots in the right column at each time point (blue = control AMC, gray = control + SS/TS, red = mutant AMC, yellow = mutant + SS/TS). Principle component analysis is used for dimensionality reduction to assess the overall contribution of the SS/TS procedure to craniofacial growth (**f**; group A = control AMC, group B = mutant AMC, group C = control + SS/TS, group D = mutant + SS/TS). Principle component 1 (PC1) is a major clustering driver, plotted for each treatment group as a function of intervention time (**g**). *P < 0.05, **P < 0.01, ***P < 0.001, ****P < 0.000 1. All data are reported as mean ± standard deviation and represent a minimum sample size of *n* > 4. Statistical analysis was carried out in GraphPad Prism v10. Student’s *t* test and two-way ANOVA were used to determine the statistical significance of observed values between experimental groups, where *P* < 0.05 was considered significant. Tukey’s test was used as a single-step method to determine differences between group means, compare multiple means, and contrast statistical significance between groups. Male and female mice were included in all analyses unless explicitly stated otherwise

Our data suggested that implantation of an engineered stem cell niche during growth and development could reduce the severity of craniosynostosis, which we aimed to assess in the *caBmpr1a* mouse model. We designed three interventions at P21, P28, and P42, all occurring after suture fusion (and weaning) but at various stages throughout the weaning-stage of development, before the completion of skull growth (Fig. 7a, b). We successfully performed the surgical suturectomy and triphasic scaffold implantation (SS/TS) procedure, including the transplantation of autologous BMSCs, in control and *caBmpr1a* mutant mice as young as P21 (Fig. S7A, B). Four weeks after SS/TS was performed, we assessed craniofacial phenotype by microcomputed tomography and landmark analysis. The triphasic scaffold maintained a patent engineered suture in each intervention cohort, and no re-synostosis was observed (Fig. 7c-e). In each intervention group, age-matched non-surgery controls (of both control and mutant genotype) are used for comparison to determine the degree of normalization of craniofacial growth as a function of intervention time.

In the P42 intervention cohort, no overt differences were observed between the control age-matched cohort (AMC) and control SS/TS mice after four weeks (Fig. 7c, Fig. S8A, Table S6A). Comparing Mutant SS/TS mice to Mutant AMC mice, the SS/TS reduced the severity of the craniosynostosis phenotype by a significant difference in 10 of 49 measurements. However, treated mutant mice are still statistically differentiated from control AMC. Compared to Mutant AMC mice, the SS/TS treatment caused an increase in the overall skull length (21.29 ± 0.76 mm vs. 20.17 ± 0.46 mm), anterior skull length (5.60 ± 0.29 mm vs. 4.35 ± 0.60 mm), nasal bone length (6.58 ± 0.10 mm) vs. 4.96 ± 0.577 mm), anterior width ratio (W7/L1, 0.369 ± 0.012 vs. 0.501 ± 0.055) and posterior width ratio (W11/P, 0.553 ± 0.013 vs. 0.585 ± 0.010).

In the P28 intervention, a more noticeable dampening of the disease burden is observed in the craniofacial phenotype of *caBmpr1a* mutant mice treated by SS/TS after four weeks, as indicated by the difference between the red and yellow lines in the spider plot shown at right (Fig. 7d, Fig. S8B, Table S6B). Like the P42 cohort, no overt differences between Control AMC and Control SS/TS mice are observed. Critically, we observed that mutant SS/TS mice significantly differ from Mutant AMC mice in 14 of 49 measurements, indicating enhanced efficacy of our intervention at this stage. Compared to Mutant AMC mice, the SS/TS treatment caused an increase in the overall skull length (20.20 ± 0.24 mm vs. 19.34 ± 0.59 mm), anterior skull length (5.23 ± 0.09 mm vs. 4.36 ± 0.43 mm), nasal bone length (5.97 ± 0.21 mm vs. 4.88 ± 0.67 mm), anterior width ratio (W7/L1, 0.382 ± 0.014 vs. 0.500 ± 0.060) and posterior width ratio (W11/P, 0.551 ± 0.037 vs. 0.570 ± 0.0.054).

In the P21 intervention, we observed the most significant therapeutic effect of the SS/TS after four weeks (Fig. 7e, Fig. S8C, Table S6C). In the spider plot shown at right, the yellow line tracks more closely with the blue (control AMC) line than the red (mutant AMC), indicating significant rescue of the craniofacial phenotype. Compared to Mutant AMC mice, the SS/TS treatment caused a significant change in the anterior skull length nearer to Control AMC (5.81 ± 0.535 mm Mutant SS/TS, 4.763 ± 0.592 mm Mutant AMC, 6.34 ± 0.120 mm Control AMC), nasal bone length (6.45 ± 0.685 mm Mutant SS/TS, 5.37 ± 0.626 mm Mutant AMC, 7.08 ± 0.193 mm Control AMC), and anterior width ratio (W7/L1, 0.390 ± 0.024 Mutant SS/TS, 0.468 ± 0.042 Mutant AMC, 0.347 ± 0.020 Control AMC).

To assess efficacy, we performed principal component analysis, controlling for the age at intervention (Fig. 7f). Considering 49 morphometric features, PCA revealed distinct variation patterns associated with genotype and the effects of surgery. PC1 and PC2 capture 58.86% of the variance (Table S7). The PCA scores plot illustrates a separation of four distinct clusters. Mutant SS/TS mice form a distinct cluster separate from Mutant AMC, indicating that the surgical intervention directly impacts skull shape in *caBmpr1a* mutant mice. In contrast, Control SS/TS mice overlap considerably with control AMC, as expected, indicating no adverse effect of the intervention. In a longitudinal analysis of the first principal component (PC1) as a function of time, we showed an inverse relationship of effect size with age, confirming that the time of surgical intervention significantly affects the resulting phenotype of the mutant mice (Fig. 7g).

**Fig. 8:**
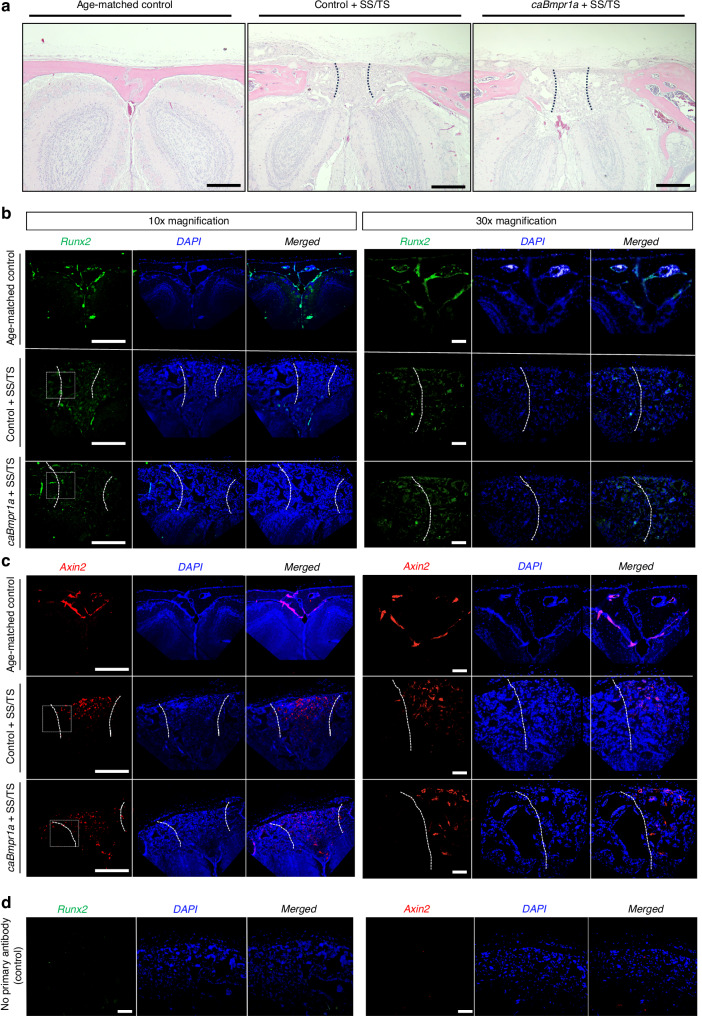
Triphasic scaffold regionally maintains cranial suture stem cells. Weaning-stage control and caBmpr1a mice were subjected to treatment at P21 (3 weeks) by surgical suturectomy and triphasic scaffold implantation (SS/TS). Histologic assessment after 4 weeks is performed using hematoxylin and eosin stain. Scale bar = 500 µm (**a**). Immunohistochemistry for RUNX2 (**b**) and AXIN2 (**c**) in coronal sections of the defect site is used to assess osteogenic potential (RUNX2) and maintenance of stemness in the suture niche (AXIN2). Differential distribution of AXIN2 demonstrates the triphasic scaffolds’ ability to support a cranial suture-mimicking tissue, compared to age-matched control mice. 10× magnification shown (scale = 200 µm). **d** Isotype controls with no primary antibody are acquired at 30× magnification with exposure settings the same as experimental sections, in age-matched control mice, processed in parallel (scale = 200 µm)

Following implantation in vivo, we sought to assess the histologic outcomes of the engineered suture niche and assess the triphasic scaffold’s ability to recapitulate stem cells as seen in the native wild-type suture. Histologic observation by hematoxylin and eosin staining (Fig. 8a) indicates that the triphasic scaffolds are well cellularized and integrated. Contrary to the large pore scaffold (Fig. S6A), no signs of ectopic bone formation are observed inside the triphasic scaffold. Given that the P21 intervention group demonstrated the most robust efficacy after 4 weeks, we assessed expression of RUNX2 and AXIN2 by immunohistochemistry. RUNX2 -positive cells are observed throughout the triphasic scaffold in both control and caBmpr1a mice, mirroring the expression of RUNX2 in both the suture and calvarial bone of non-treated control mice (Fig. 8b), suggesting that the scaffold supports osteogenic lineage commitment. On the other hand, the triphasic scaffold demonstrates a capacity to augment AXIN2 -positive cells specifically within its central small-pore region (Fig. 8c). The persistence of AXIN2 -positive cells within the central, non-ossified portions of the scaffold indicates the maintenance of a stem cell pool, which is essential for suture patency and regenerative capacity.

## Discussion

The cranial suture houses vital skeletal stem cells that facilitate growth, development, and bone repair. In craniosynostosis, these cells are lost prematurely, causing early suture fusion and serious developmental sequelae that are not fully resolved by current surgical care standards. A tissue engineering approach to treating craniosynostosis is promising to reimplant and reestablish the suture skeletal stem cell (SSPC) population, particularly for patients with unknown disease etiology (~80%^52^), to improve the efficacy of surgical suturectomies and re-establish skull growth.^53^ The objective of this study was to test whether a regenerated skeletal stem cell niche can normalize craniofacial growth during the weaning-stage growth period when cranial dysmorphology becomes permanent. Because the scaffold is biodegradable and not intended as a permanent prosthesis, restoration of normal developmental trajectory—rather than indefinite patency—is the translational success criterion. Here, we report an effective treatment in which a biomaterial scaffold selectively maintains the stemness of autologous MSCs, thereby optimizing its ability to recapitulate the cranial suture stem cell niche. We provide robust in vivo evidence to suggest that the triphasic scaffold can support cranial suture regeneration and correct skull dysmorphology in caBmpr1a mice with craniosynostosis during a 4-week timespan, bridging the critical period of craniofacial growth and development. These findings have significant potential clinical value.

Biomaterial scaffolds serve as an artificial extracellular matrix to support the spatial organization of cells within a defect.^54–56^ Hong and Mao demonstrated transplanting dermal fibroblasts on a gelatin scaffold into a rabbit bone defect, creating an “artificial suture.” In contrast to a rhBMP2-loaded collagen sponge, the fibroblast-gelatin construct favored fibrous tissue over radiographic bone formation after four weeks.^27^ Subsequently, the same group used a collagen sponge loaded with BMSCs and TGFβ3 to delay suture fusion by suppressing bone formation.^57^ More recently, Yu et al. demonstrated a high-density Matrigel-GelMA to replace a fused coronal suture in *Twist1*^*+/-*^ craniofacial anomalies, a mouse model mimicking Saethre-Chotzen syndrome.^28^ Their Matrigel-GelMA biomaterial composition was chosen to suppress bone development by preventing diffusion of signaling molecules, diffusion of blood, and blood vessel ingrowth. Rather than fabricating a stem cell niche in isolation (i.e., small pore monophasic), our triphasic scaffold approach designed a niche that integrates within the calvarial bone microenvironment, taking advantage of the critical role of pore size and curvature in macroporous scaffolds to guide MSC fate.^34,35,37^ Rather than occluding cell migration or tissue maturation with a high-density matrix, multiple compartments lend to host integration between tissue compartments with distinct phenotypes. Critical to its design, the triphasic construct allows differentiation and maintenance of stemness in a single implantable construct where cell migration is required for suture function.^58^ We demonstrated that the triphasic scaffold provides a passive process of differential migration to select for maintenance of stemness in its center region and osteogenic differentiation in the flanking region. Few naïve BMSCs migrate from the center pore region; we believe those that do migrate at later time points (e.g., 2 weeks) are undergoing differentiation.

The mechanism for this phenomenon is unclear and remains an interesting topic for future mechanistic studies. In the HoxA11 lineage tracing model, the number of RFP-positive cells appears lower in the monophasic versus triphasic scaffolds, despite maintaining the same cell seeding density. Given that the large pore motif promotes cell migration, it is not an unexpected finding that the number of RFP^+^ cells decreases in the large pore scaffold over time. In this experiment, we are specifically interested in the correlation between GFP^+^; RFP^+^ and RFP^+^ cells to understand the ability of our scaffolds to maintain stem cells within the scaffold. We propose two potential contributing factors. First, our previous studies demonstrated that pore size differentially controls YAP/TAZ signaling,^38–40^ where >250 µm pores facilitate marked upregulation of YAP activation. YAP/TAZ activity is necessary for persistent cell migration. Depletion of YAP/TAZ impairs cell motility, as observed in <125 µm pores, reduces migration speed, and leads to migratory arrest, while their activation enables cells to remodel their cytoskeleton and focal adhesions, supporting ongoing movement.^59–61^ Second, the triphasic scaffold creates a clear gradient of vasculature. SDF-1 (stromal cell-derived factor-1)/CXCR4 axis is a primary pathway guiding BMSC migration toward blood vessels. SDF-1 is secreted by endothelial and other tissue cells, creating a gradient that attracts BMSCs expressing the CXCR4 receptor.^62,63^ Similarly, other chemokines and growth factors involved include PDGF, TGF-β1, VEGF, and FGF2, which further enhance activated MSC migration and homing to vasculature as an important step in osseous wound healing.^64,65^

The *caBmpr1a; Wnt1*-Cre mouse model demonstrates the triphasic scaffold’s therapeutic potential, as these mutant mice develop a midline craniosynostosis phenotype similar to human patients. This model expresses the constitutively active BMP type 1A receptor (caBmpr1a), enhancing BMP signaling and shifting cranial neural crest cells (NCCs) toward endochondral ossification, resulting in premature cranial suture fusion and skull dysmorphology.^22^ The craniofacial phenotype in caBmpr1a mice is fully established during the weaning-stage growth window, after which skull morphology becomes biomechanically fixed. Therefore, reestablishing stem cell dynamics only during this defined period is sufficient to permanently redirect craniofacial shape, even if the endogenous suture tissue later matures. This concept parallels clinical suturectomy timing, where durable correction arises from transient restoration of normal growth vectors rather than persistent suture patency.

We showed that our suturectomy procedure is safe and minimally traumatic; both control and caBmpr1a mutant animals recover well. The suturectomy or suturectomy with monophasic large pore scaffold alone suffices for causing re-synostosis of a previously unfused suture. Conversely, the three post-weaning interventions with the triphasic scaffold effectively normalized the craniofacial phenotype of caBmpr1a mice to match the control. The PCA clustering highlights the significant phenotypic differences influenced by genetic and surgical factors on the adult craniofacial phenotype. At earlier time points (3- and 4-week intervention), PC1 values for mutants treated with SS/TS are closest to control values, indicating that earlier interventions make the skull shape of mutant mice more similar to the control group following SS/TS. Cranial neural crest cells may be primed by increased BMP signaling before birth, influencing their perinatal behavior in craniofacial development. Thus, earlier intervention, near the weaning-stage, offers more significant disease phenotype relief than later development. This early postnatal growth window in mice parallels the infant–toddler period in humans, during which surgical intervention for craniosynostosis is clinically indicated to redirect craniofacial growth.

Following implantation in vivo, we evaluated whether the engineered construct recapitulates key features of a suture-like microenvironment. Histologic analysis demonstrates that the triphasic scaffold is well cellularized and integrated, and no ectopic bone formation is observed within the central region over the four-week observation period. Immunostaining further indicates that *Axin2*^*+*^ cells are preferentially maintained within the central small-pore compartment, consistent with preservation of a suture-associated stem/progenitor pool,^20^ while *Runx2*^*+*^ cells are present throughout the engineered tissue, supporting osteogenic lineage competence within the construct. The scaffold’s ability to selectively enrich Axin2-positive cells within its central small-pore region highlights its potential to maintain and expand the suture stem cell population, a critical feature for successful niche regeneration. Together with the pore-dependent organization of vascularization, these findings support the notion that the triphasic scaffold creates a spatially patterned microenvironment consistent with a functional “bone–suture–bone” architecture during the weaning-stage growth window.

A key objective of this study was to test whether temporally limited restoration of a suture-like microenvironment during the weaning-stage craniofacial growth period is sufficient to reduce the disease burden in a craniosynostosis model. Because craniofacial morphology becomes largely fixed after the weaning-stage craniofacial growth period, durable correction can arise from redirecting growth trajectory during a finite developmental window, rather than requiring indefinite persistence of an engineered suture niche. Longer-term evaluation of scaffold remodeling after degradation and targeted lineage-level mechanistic studies to determine the degree of autonomous niche regeneration remain important future directions but are beyond the scope of this proof-of-concept study focused on developmental trajectory rescue.

Our study identifies an optimal window for safe early intervention at 3-4 weeks, addressing key questions raised by prior suture regeneration attempts.^28^ These findings endorse the triphasic scaffold to prevent resynostosis and maintain a skeletal stem cell niche that can contribute to bone formation over time. In addition, we demonstrated the feasibility of a piezoelectric handpiece over a traditional rotary instrument, highlighting significant advantages for minimally traumatic procedures.^66–68^ Due to their ultrasonic frequency, piezoelectric devices cut mineralized tissue while preserving soft tissues like nerves and blood vessels. This surgery causes less soft tissue trauma, reduces postoperative pain and swelling, and offers better temperature control than rotary instruments,^68^ which holds significant potential for less invasive treatment.

Cell sourcing is a key consideration for the clinical implementation of this tissue-engineering treatment for human patients. Previous reports have isolated and characterized mesenchymal stem cells from the suture (SMSC).^9,11,20,34,35^ Obtaining suture cells from a human patient for autologous transplantation presents challenges due to morbidity, limited cell quantity, and identifying sutures unaffected by mutations. This work demonstrates the utility of autologous mesenchymal stem cells from bone responsive to the biomaterial microenvironment without costly in vitro conditioning. BMSCs and SMSCs are multipotent cells capable of maintaining stemness or differentiating into osteogenic, chondrogenic, and adipogenic pathways. However, BMSCs with a high progenitor cell concentration are more easily obtained in large quantities, minimizing hemoglobin loss^69^ and lessening the need for extensive in vitro expansion and conditioning, which may aberrantly affect stem cell character.^70,71^ There are valid questions on how long BMSCs can survive in the suture niche and whether there are any long-term adverse impacts for skull growth warranting future studies. Our approach, however, is generalized for this large patient population due to biomaterial microenvironments’ influence on cell differentiation and migration defect rather than mutation or syndrome-specific.

In summary, our findings validate a newly designed biomaterial scaffold that successfully recapitulates a skeletal stem cell niche in vivo and demonstrates its efficacy in a regenerative treatment for craniosynostosis. Current treatment options for affected pediatric patients are severely limited and do not address the disease’s underlying causes. A cell-scaffold construct that recapitulates the cranial suture mesenchyme and surrounding bone, collectively comprising the suture niche, presents an opportunity to enhance patients’ long-term quality of life and reduce the disease’s developmental and neurological sequelae. The latter is of great interest and will be evaluated in future studies, given the recent finding that temporal treatment significantly improves skull deformity, and neural behaviors associated with mutations in Twist.^28^ Our findings indicate that an implanted engineered suture creates a skeletal stem cell niche that preserves stem cells and facilitates their contribution to craniofacial growth. Longer-term studies are critical to evaluate the persistence and remodeling of the engineered tissue after scaffold degradation; however, such studies are beyond the goals and scope of our proof-of-concept, which was designed to assess regenerative redirection of growth during the developmental window when craniofacial dysmorphology becomes permanent.

## Materials and methods

### Experimental design

Power analysis determined the sample size to achieve a reliable measurement of the effect. Data collection in animal experiments occurred at predefined endpoints to assess therapeutic intervention after 4 weeks. All experiments were performed in biological and statistical replicates, as described below. The overarching research objective is to determine the effects of the triphasic scaffold on the stemness of skeletal mesenchymal stem cells and determine its therapeutic efficacy as a surgical intervention in a mouse model of craniosynostosis. Both healthy (control) and disease (craniosynostosis, caBmpr1a) mice were used. Mice were randomly assigned to intervention or age-matched control groups and intervention cohorts; no data were excluded from the analysis. An equal number of male and female mice were used. Efficacy was assessed by microcomputed tomography, morphometric analysis, and histologic observations. A calibrated examiner, blinded to the intervention, performed a quantitative morphometric analysis.

### Triphasic scaffold fabrication

Large pore monophasic nanofibrous, macroporous tissue engineering scaffolds were fabricated from poly (L-lactic acid) (PLLA) based on an established method.^43^ Sugar spheres were packed into the mold in three phases to fabricate the triphasic scaffold. First, 250–425 µm spheres were deposited to 2–4 mm and annealed for 3 min at 37 °C. The purpose of this step is to prevent subsequent sugar layers from penetrating into this step. Next, 60–125 µm spheres were added to 0.5–2 mm and annealed for 2 min at 37 °C. Finally, hexane covered another layer of 250–425 µm spheres, which were annealed for 6 min at 37 °C. The templates were dried under a vacuum to remove hexane. PLLA (10% wt/v in THF) was cast and cooled to -80 °C for 48 h to induce phase separation via TIPS. Constructs were then transferred to hexane for 24 h and soaked in water for 24 h to eliminate the sugar porogen. The result is a three-dimensional, macroporous tissue scaffold that can be cut to size with a biopsy punch. Morphology was examined by scanning electron microscopy. Before in vitro and in vivo work, PLLA scaffolds were sterilized with ethylene oxide gas per the manufacturer’s protocol (Anpro). They were washed with 70% ethanol for 30 min, followed by PBS four times, and serum-free media before seeding. These washing steps prepared a hydrophilic surface for better cell adhesion.

### Scanning electron microscopy (SEM)

SEM imaging evaluated the surface topographies of PLLA scaffolds at nano and micro scales. Scaffolds were attached to sample holders with double-sided carbon tape, gold-coated for 120 s, and observed at 5 kV with a 10 mm working distance (JEOL JSM-7800FLV).

### Cell and animal experiments

Mice were deemed healthy before procedures for all animal experiments. Littermates were randomly assigned to experimental groups; littermates genotyped negative for mutations were used as hosts, age-matched control (AMC), and cell donors as “control” mice. Genotypes are confirmed three times. Both male and female mice were used. All studies followed guidelines set forth by the University of Michigan Institutional Animal Care & Use Committee (IACUC) under an approved protocol (PRO00011263) and conducted according to ARRIVE guidelines. Animals were maintained in group housing at 72 ± 2 °F in ventilated cages. Animals were euthanized for experiments by CO2 exposure, followed by bilateral pneumothorax and organ retrieval. All surgical procedures were performed under general anesthesia according to guidelines approved and issued by the IACUC. Post-operative pain was controlled using carprofen (5 mg/kg SC/IP); mice were monitored daily for 7–10 days following the surgical procedure and twice weekly after that. Details of each mouse model and surgical interventions are provided in the Supplementary Methods.

### BMSC isolation

According to Maridas et al., primary bone marrow stromal cells (BMSCs) were isolated from the femora and tibiae of 4- to 6-week-old mice and maintained in Dulbecco’s Modified Eagle Medium (DMEM) containing 10% FBS. All cells were used for experiments before passage 4.^72^

### Craniosynostosis model

Mice carrying the Cre-inducible constitutively activated Bmpr1a (*caBmpr1a*) transgene (B6;129S7-Tg(CAG-lacZ,-BMPR1A*,-EGFP)1Mis/Mmjax, 032018-JAX) was described previously.^24^ caBmpr1a were bred with Wnt1-Cre mice (B6.Cg-Tg(Wnt1-cre)11Rth Tg(Wnt1-GAL4)11Rth/J, Jackson Lab, Stock No. 009107), resulting in a distinct craniosynostosis phenotype.^22^ “Mutant” mice carried both *caBmpr1a* and *Wnt1-Cre*; “control” mice carried *caBmpr1a*, but not *Wnt1-Cre*. This genotype shows no overt phenotype.^22,24,29,31^

### Surgical suturectomy model

A surgical strip suturectomy model was used to assess the function of triphasic scaffolds to replace the cranial suture and establish an engineered skeletal stem cell niche in vivo. This procedure was safely performed on mice as young as P21 and as old as P84 in male and female mice. *Wnt1-Cre*(-) (“control”) and *caBmpr1a; Wnt1-Cre*(+) (“mutant”) mice were anesthetized by isoflurane gas and the surgical site was prepared for surgery. A single 5–6 mm incision along the cranial vault was made, and the periosteum was displaced. A piezoelectric surgical handpiece (Piezosurgery Flex, Mectron s.p.a.) with a diamond bur (MF4, MF5 – Mectron s.p.a.) was used to make a full-thickness osteotomy along the metopic suture using copious irrigation. The defect extended from 0.5 mm posterior to the frontonasal suture to the midpoint of the metopic suture at the narrowest region of the frontal bone, yielding a defect 3.0–3.5 mm in length, 1.2–1.5 mm in width, and total thickness (approx 0.8–1.0 mm). The full-thickness defect was made while respecting the underlying dura mater and brain tissue. Scaffolds were stabilized within the defect site, and the overlying tissue was closed with 5-0 Vicryl sutures. Post-operative pain was managed with carprofen (5 mg/kg, SC/IP) for 48 h. No neurologic defects were observed clinically or histologically.

### Statistical analysis

All data are reported as mean ± standard deviation and represent a minimum sample size of *n* > 4. Statistical analysis was carried out in GraphPad Prism v10. Unless otherwise described, in the case of morphometric analysis, student’s *t* test and two-way ANOVA were used to determine the statistical significance of observed values between experimental groups, where *P* < 0.05 was considered significant. Tukey’s test was used as a single-step method to determine differences between group means, compare multiple means, and contrast statistical significance between groups. Male and female mice were included in all analyses unless explicitly stated otherwise. Statistical analyses were carried out under the guidance of the University of Michigan Consulting for Statistics, Computational and Analytical Research Center (CSCAR). All graphics note significance as: *P < 0.05, **P < 0.01, ***P < 0.001, ****P < 0.000 1.

### Picrosirus red image analysis

Picrosirus red (PSR) stained images were imaged with an Olympus BX51 microscope and a digital camera equipped with a polarized light filter, and digital image analysis was carried out according to our previously published method.^73^ A minimum of *n* = 10 images were analyzed in each group.

### CD31 vascularization image analysis

CD31 analysis of immunohistochemistry prepared sections was carried out according to our previously published method.^73^ Briefly, TIF images were acquired using a digital camera attached to a microscope. For the CD31^+^ area, Fiji was used to selectively threshold the CD31^+^ area using a mask, but not cell nuclei or background, and measured it. A minimum of *n* = 20 images were analyzed in each group. Raw images (.TIF) were scaled appropriately for blood vessel diameter to convert pixel distances to microns. Then, the diameter of each blood vessel was measured as a straight line across the widest part. Only round blood vessels (i.e., perpendicular to the section) were measured. A minimum of *n* = 90 measurements were made across a minimum of *n* = 10 images per group.

### Cell migration analysis

Prior to cell seeding, scaffold constructs were assessed for width under a microscope to ensure quality and confirm 1 mm height. To assess in vitro cell migration 300 000 cells were seeded uniformly, or 100 000 cells seeded to the center region only (representing 1/3 the width of the construct, to maintain cell seeding density). Cells were seeded at a density of 20 000 cells/µL in order to use a minimum volume of media. Cells were allowed to adhere to the scaffold for 30 min at 37 °C, then additional culture media was added. We acquired z-resolved confocal laser micrographs of cell-scaffold constructs at each time point in order to observe cells across the entire 1 mm depth of the scaffold. Image analysis segmentation was used to identify the x-y-z coordinates of each cell, restricted by size and threshold to avoid background signals. Dimensionality was minimized in the z-direction, and cell migration is assessed as a function of the x-y coordinate along the long axis of the scaffold. Bright-field images acquired simultaneously were used to establish the small-large boundary. Analysis was performed blinded.

### Colocalization analysis

Bright field images were used to determine the boundary between small and large sections of the scaffold, informing the region of interest (ROI) selection. ROI was held constant between channels of the same section, and chosen from the bright field image of each section. Colocalization threshold analysis is carried out in Fiji according to the Costes method auto threshold determination, which removes bias of threshold selection and visual interpretation by simultaneously estimating the maximum intensity threshold for each color below which pixels do not show any statistical correlation.^49^ A linear regression solution is extrapolated, plotting the RFP signal along the y-axis and GFP along the x-axis. Pearson’s correlation coefficient above the threshold is calculated as Rcolocalization. The degree of colocalization is assessed by Mander’s method to compute the thresholded Mander’s split colocalization coefficient,^50^ where zero is no colocalization, and one means perfect colocalization. The analysis is repeated on *n* = 5 samples per group per time point and *n* > 4 sections (images) per sample.

### Bone fill profile analysis

Micro-CT reconstructed sections in 2D were constituted along the axis perpendicular to the long axis of the specimen (skull, defect, scaffold). The threshold was adjusted accordingly for trabecular bone identification. Serial sections were obtained and exported across the defect site. The signal intensity profile was assessed in Fiji across the defect site in a standardized rectangular ROI matching the width and depth of the clinical defect. The analysis is repeated on *n* > 4 samples per group per time point and *n* > 4 sections (images) per sample. Analysis was performed blinded.

### Morphologic analysis

Reconstructions from micro-CT image acquisition were converted to 3D volumes, and landmarks were placed using Dragonfly (ORS, Denver, USA, v.2022.2). All landmarks were digitized by the same calibrated investigator with a set of 32 landmarks in a blinded fashion. The landmarks are shown as six subsets of measurements in Table 1 and Appendix Figure 15. Our method allows us to appreciate how different regions of the craniofacial skeleton are growing in proportion to the overall A-P dimension. Since the scans of each animal were not always obtained with teeth in occlusion, the mandible was analyzed separately using Euclidean distances. We calculated the distance between two points using (x, y, z) coordinates. The angle subtended by three consecutive points was calculated using the law of cosines and vector dot product. All calculations were performed using a Microsoft Excel macro built by our group. Principle component analysis was carried out using a standardization method. Using Monte Carlo simulations, principal components (PCs) were selected based on parallel analysis to calculate all resulting PCs’ eigenvalues. PCs with eigenvalues greater than those from simulations at the 95th percentile were set after 1 000 simulations. Statistical analysis for each trial was carried out for all samples using the Krustal-Wallis test to determine significant differences across multiple variables within and across groups and time points. All morphometric data were analyzed using nonparametric tests to avoid assumptions about variance or distribution. We corrected for multiple comparisons by controlling the false discovery rate (FDR) using the two-stage step-up method of Benjamini, Kreiger, and Yekutieli, with a desired FDR of 0.05. These analyses were performed using Prism (v10, GraphPad 2023).

### Immunohistochemistry

Paraffin-embedded tissue sections underwent deparaffinization and rehydration through graded xylene and ethanol washes, followed by post-fixation in 10% formalin and a hydrogen peroxide treatment to block endogenous peroxidase activity. Antigen retrieval was performed using Diva Decloaker solution at 60 °C overnight. After rinsing and blocking in PBS containing 0.1% Triton X-100 and 10% donkey serum, slides were incubated overnight at 4 °C with primary rabbit polyclonal antibodies against Axin2 and Runx2 (1:100 dilution). Primary rabbit polyclonal antibodies against Axin2 (Abcam, Cat. # ab32197, Lot # GR294422-2) and Runx2 (Cell Signaling, Cat. # D1L7F, Lot # 12556S) were used at a 1:100 dilution. The next day, sections were washed and incubated with donkey anti-rabbit secondary antibodies conjugated to Alexa Fluor 488 or 594 (1:200) for 2 h at room temperature, followed by PBS washes. Sections were mounted using a DAPI-containing antifade mounting medium (ProLong Gold), covered with a coverslip, and imaged with a Leica Thunder microscope. Nuclear and antigen signals were visualized and merged using the microscope’s software.

Please note, additional detailed methods are found in the supplementary information.

## Supplementary information

Supplementary Materials

41413_2026_539_MOESM2_ESM

41413_2026_539_MOESM3_ESM

41413_2026_539_MOESM4_ESM

41413_2026_539_MOESM5_ESM

41413_2026_539_MOESM6_ESM

41413_2026_539_MOESM7_ESM

41413_2026_539_MOESM8_ESM

## Data Availability

All data related to the present work is provided in the figures and supplementary information. Additional requests for information should be directed to the corresponding authors.
