# Supplementary material for: A tissue engineering approach to regenerate the cranial suture skeletal stem cell niche with a multicompartment biomaterial scaffold
Source: Bone Res. 2026 May 28;14:58. doi: 10.1038/s41413-026-00539-z (PMC13219457; doi:10.1038/s41413-026-00539-z)
Supplement: Supplementary file 1 — Supplementary Materials [file 41413_2026_539_MOESM1_ESM.pdf]

A Tissue Engineering Approach Regenerate the Cranial Suture Skeletal Stem Cell Niche with a Multicompartment Biomaterial Scaffold

W. Benton Swanson[1†*], Lindsey Douglas[1,2], Seth M. Woodbury[1,2,3], Jackson Albright[1,4], Haichun Pan[1], Maiko Omi-Sugihara[1], Miranda Eberle[1,2], Jake Herremans[1,2], Hwa Kyung Nam[5], Rafael Correia Cavalcante[1], Coral Chen[1], Peter X. Ma[1,4,6,7], Nan E. Hatch[5], Yuji Mishina[1*]

[1] Department of Biologic and Materials Science, School of Dentistry, University of Michigan; Ann Arbor, MI, USA.

[2] Department of Chemistry, College of Literature, Science and the Arts, University of Michigan; Ann Arbor, MI, USA.

[3] Department of Physics, College of Literature, Science and the Arts, University of Michigan; Ann Arbor, MI, USA.

[4] Department of Biomedical Engineering, College of Engineering, University of Michigan; Ann Arbor, MI, USA.

[5] Department of Orthodontics and Pediatric Dentistry, School of Dentistry, University of Michigan; Ann Arbor, MI USA.

[6] Department of Materials Science and Engineering, College of Engineering, University of Michigan; Ann Arbor, MI, USA.

[7] Macromolecular Science and Engineering Center, College of Engineering, University of Michigan; Ann Arbor, MI, USA.

* Corresponding authors: W. Benton Swanson, DDS, PhD, Email: wbentons@umich.edu; Prof. Yuji Mishina, PhD, Email: mishina@umich.edu

† Current Address: Department of Oral Medicine, Infection and Immunity, School of Dental Medicine, Harvard University; Boston, MA, USA.

Supplementary Materials

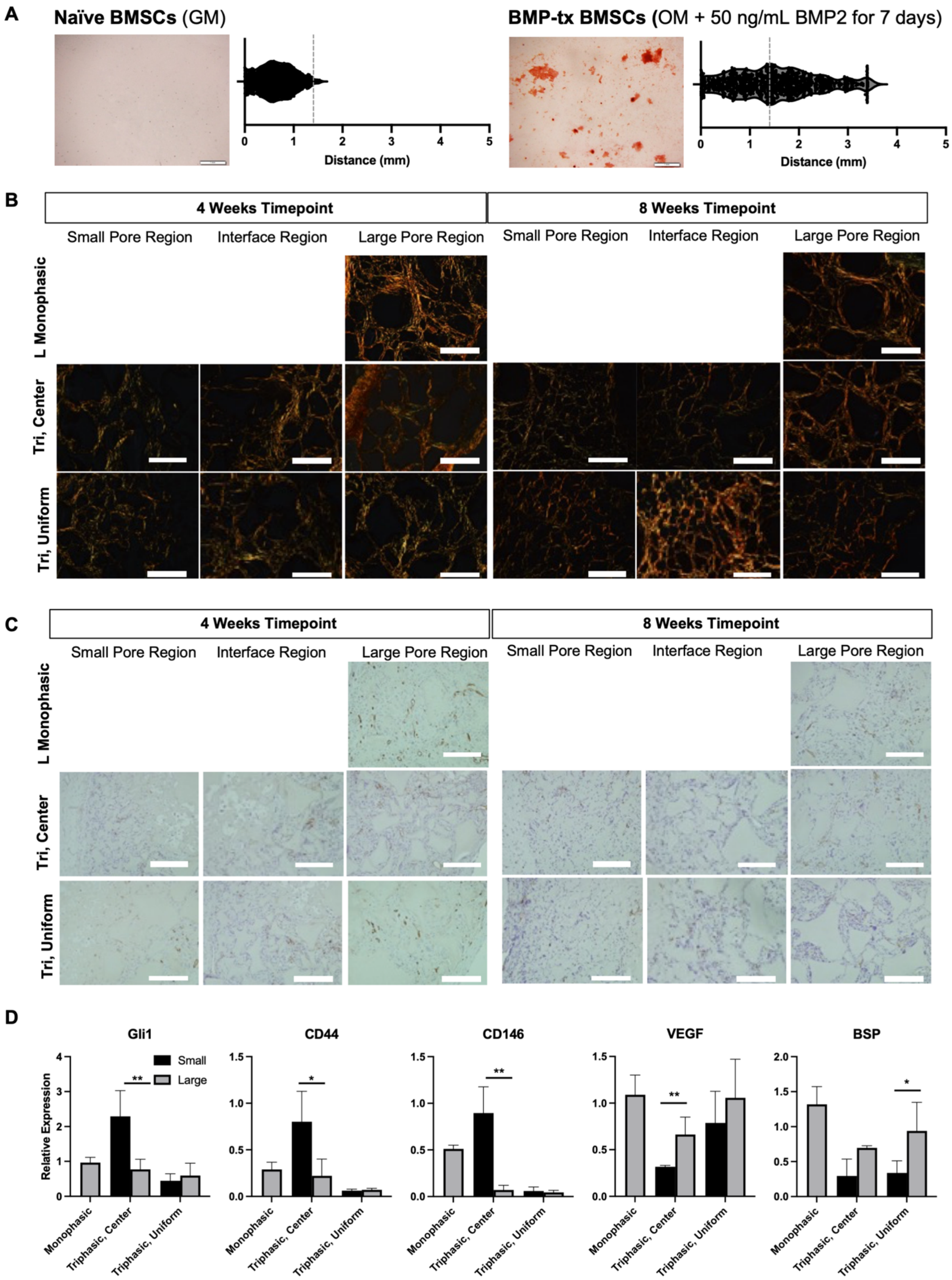

Fig S1 Naïve (cultured in growth media, GM) and differentiating BMSCs (osteogenic media, OM, with the addition of 50 ng/mL rh-BMP2) are assessed for mineralization after seven days of in

vitro culture by alizarin red staining. BMP-treated BMSCs (BMP-tx BMSCs), but not Naïve BMSCs, demonstrate mineralization as a marker of their osteogenic differentiation (**A**). Naïve or BMP-treated BMSCs are seeded to the center region of triphasic scaffolds, cultured for two weeks, then assessed by confocal laser microscopy, as shown in violin plots (n=3 biologic replicates per group). Representative histology from subcutaneous explants after 4 (left) and 8 (right) weeks, assessed by picrosirus red (**B**, scale = 500 um) and CD31 immunohistochemistry (**C**, scale = 500 um). Gene expression is assessed spatially by small (black) and large (grey) pore regions after four weeks in vivo (**D**, n=5 per group) for genes related to stemness: Gli1, CD44, CD146 and osteogenic differentiation: VEGF, BSP. * $p<0.05$, ** $p<0.01$, ***$p<0.001$, ****$p<0.0001$.

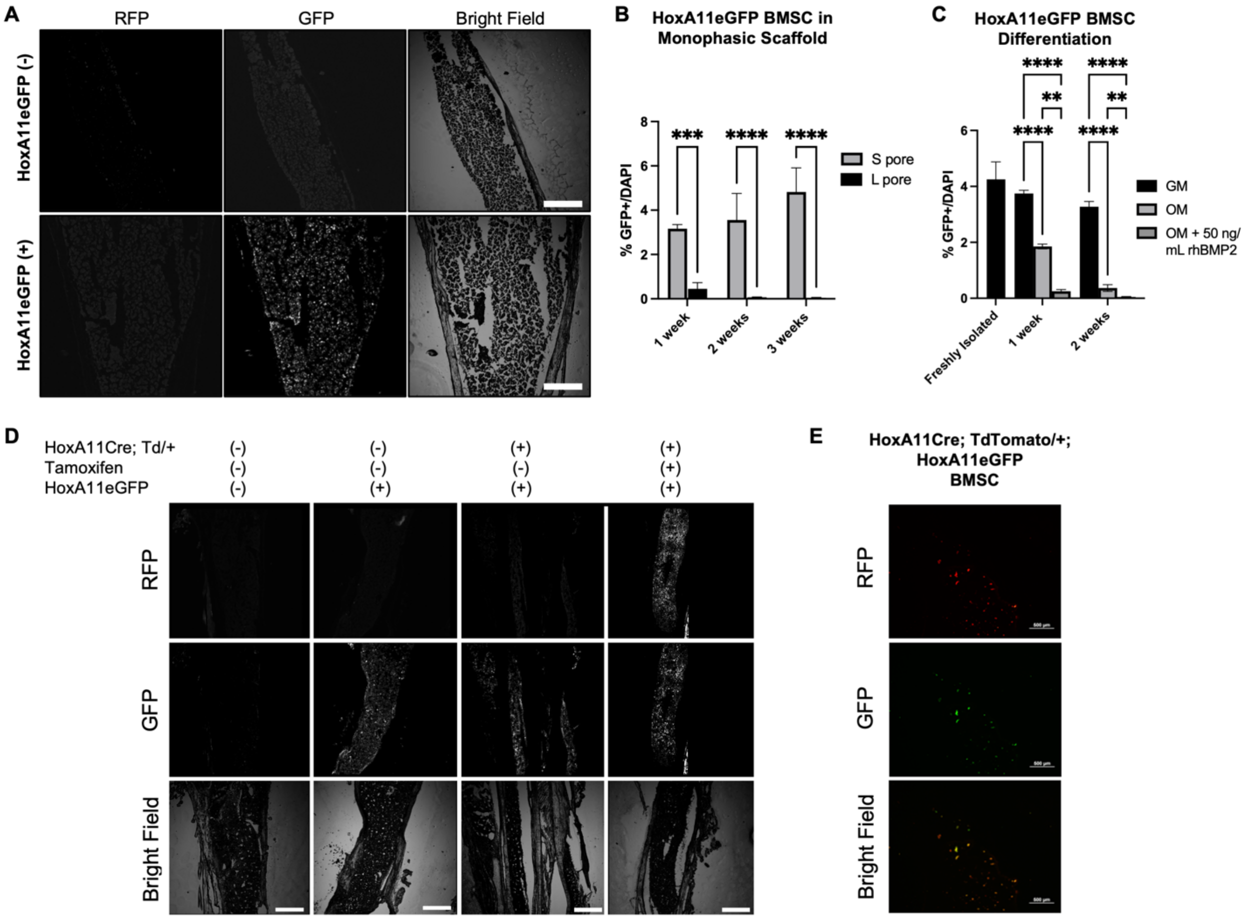

Fig S2 Histologic validation of HoxA11eGFP fluorescent transgene in vivo, in demineralized femur sections (**A**, scale = 500 um). HoxA11eGFP+ BMSCs maintain their fluorescence in small but not large-pore scaffolds (**B**). In monolayer culture, exposure to osteogenic media or osteogenic media supplemented with recombinant BMP2 (50 ng/mL rh-BMP2) induces osteogenic differentiation and decreased GFP expression (**C**). Validation of fluorescent signal in the HoxA11Cre; TdTomato/+; HoxA11eGFP mouse model in femur histology (**D**, scale = 1 mm) compared to control. This model harbors a real-time visual transgene (*HoxA11eGFP*) and Cre/loxP technology (*HoxA11Cre*^{ERT2}) to identify their progeny within the scaffold. Cre activity was activated in vivo before cell isolation to ensure its signal in marking *HoxA11*+ SSCs. BMSC isolated from the femurs of tamoxifen-induced HoxA11Cre;Td/+; HoxA11eGFP mice maintain their fluorescence at passage 3 in vitro (**E**, scale = 500 um). * $p<0.05$, ** $p<0.01$, ***$p<0.001$, ****$p<0.0001$.

Fig S3

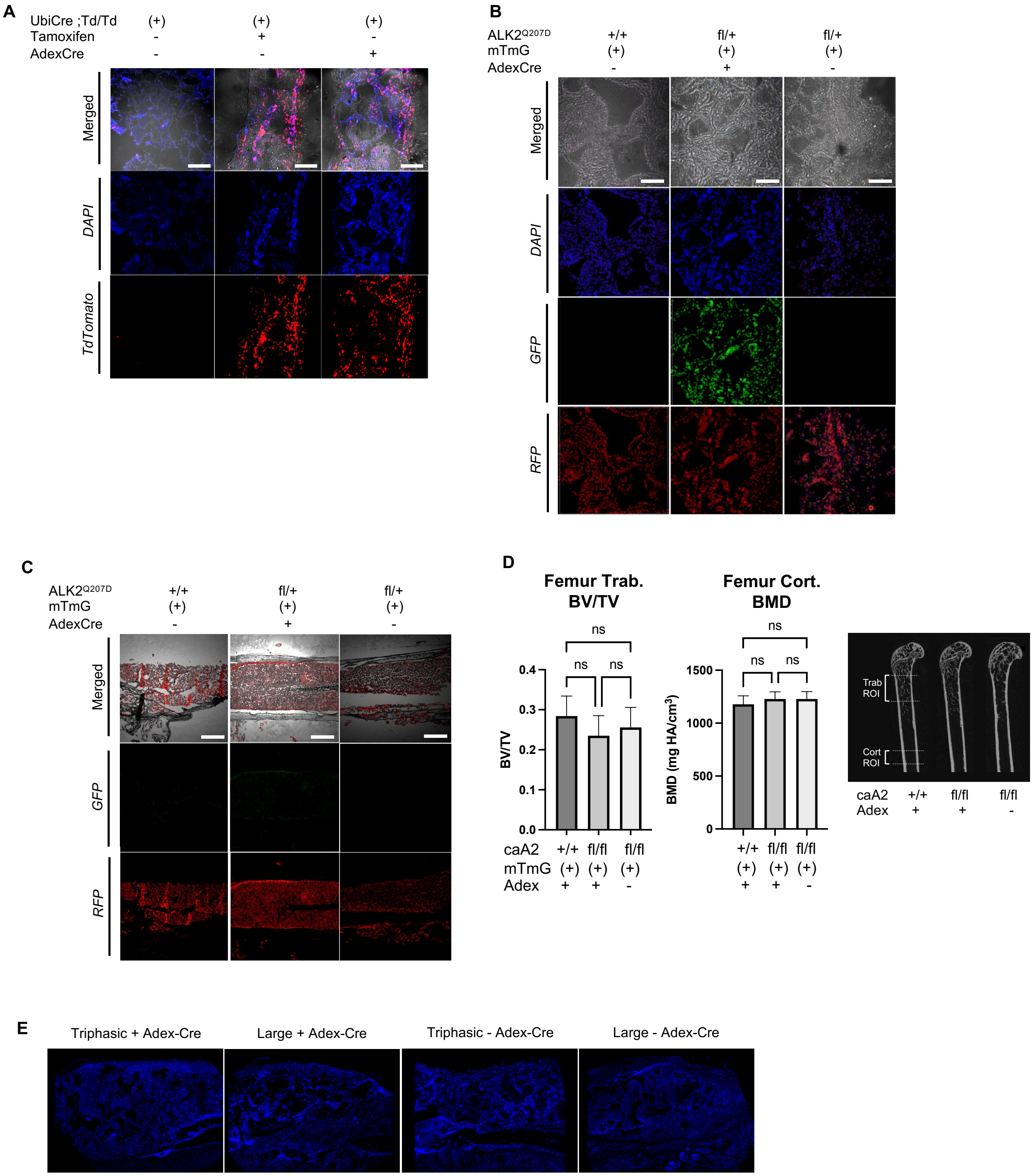

Fig S3 Transgenic fluorescent protein expression is maintained by cells migrating into subcutaneously implanted scaffolds, observed by confocal laser microscopy. In UbiCreER+;

Td/Td mouse, RFP-expressing cells are observed infiltrating the scaffold after 2 weeks with both systemic administration of tamoxifen and local administration of Adex-Cre at the scaffold site, but not in the absence of a Cre recombination inducing agent (**A,** Scale = 500 um). Adex-Cre-laden scaffolds are implanted subcutaneously in mice positive for ALK2Q207D and mT/mG to induce recombination and expression of GFP (**B**, scale = 500 um), observed by confocal laser microscopy. Despite robust recombination within the scaffold, no GFP signal is observed in the femur (**C**, scale = 1 mm), consistent with microcomputed tomography results (**D**). n=4 mice per group; representative images shown. Coronal sections of calvarial explants after two weeks in vivo are stained with DAPI to visualize cellularity and imaged by confocal laser microscopy (**E**).

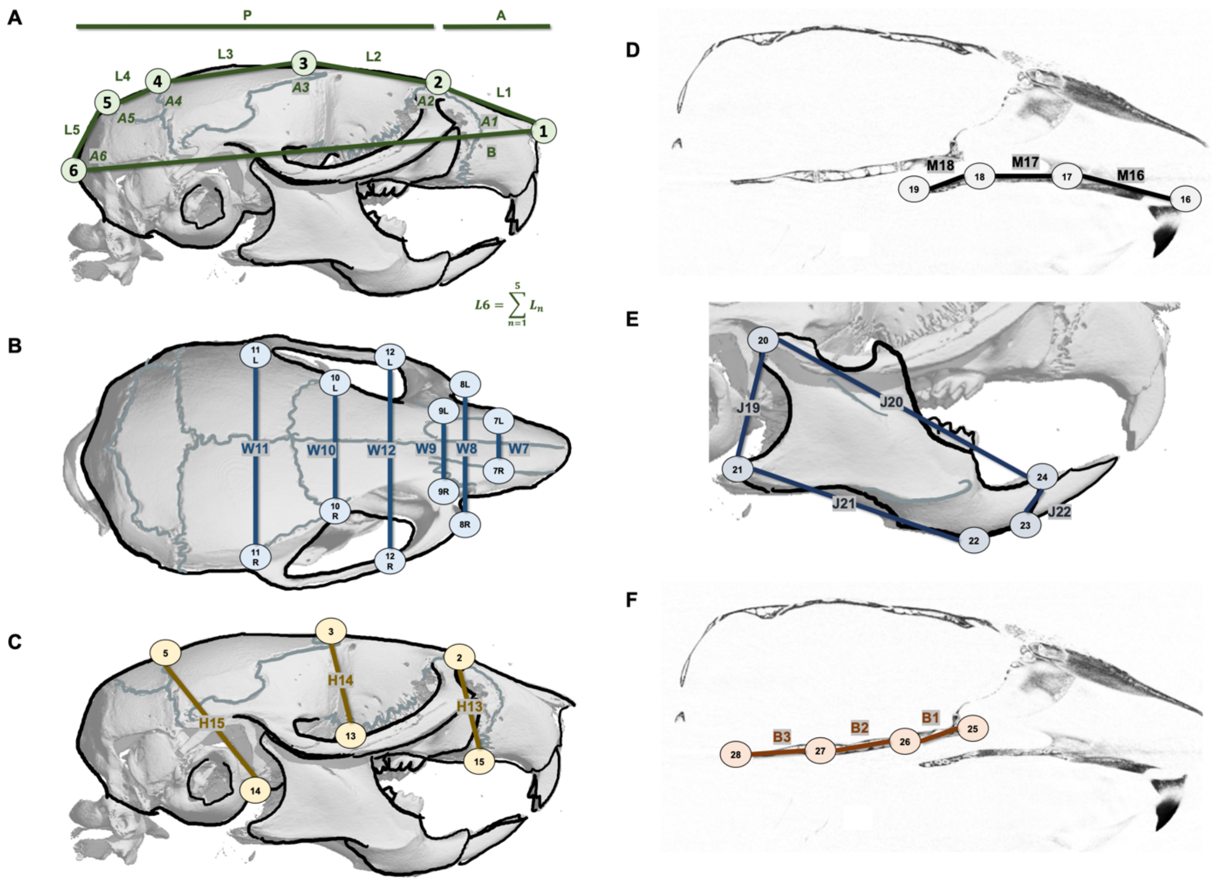

Fig S4 Morphologic landmarks used to assess craniofacial morphology and corresponding measurements. Landmarks are categorized into length (**A**), width (**B**), height (**C**), maxilla (**D**), mandible (**E**), and skull base (**F**). A list is shown in **Table 1**.

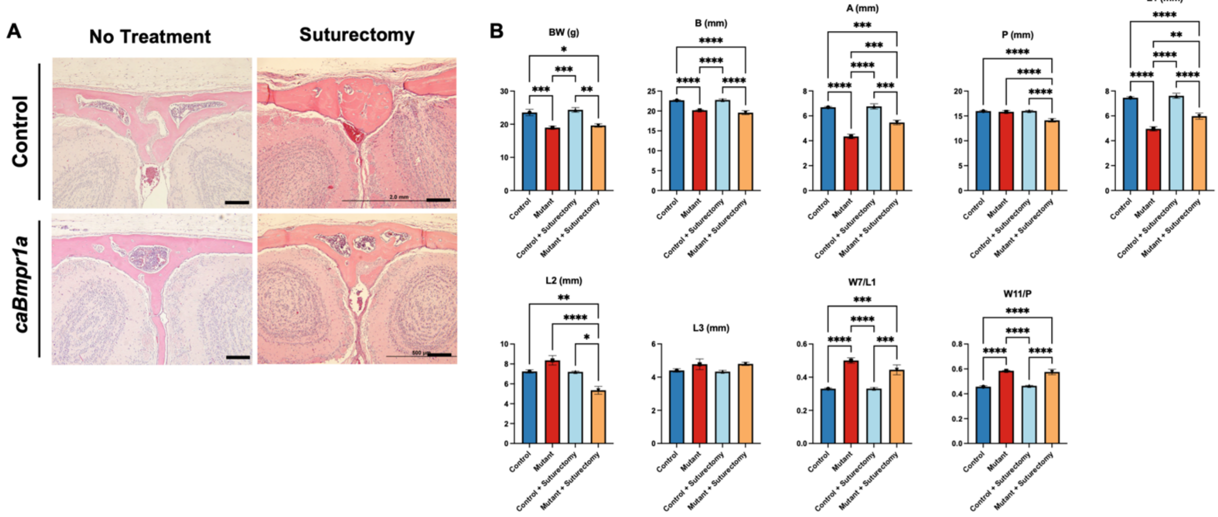

Fig S5 Representative histology (**A**), selected measurements, and descriptive statistics (**B**) describing the craniofacial phenotype of control and caBmpr1a mutant mice, treated with surgical suturectomy compared to non-treated controls, at 6W. * $p<0.05$, ** $p<0.01$, ***$p<0.001$, ****$p<0.0001$.

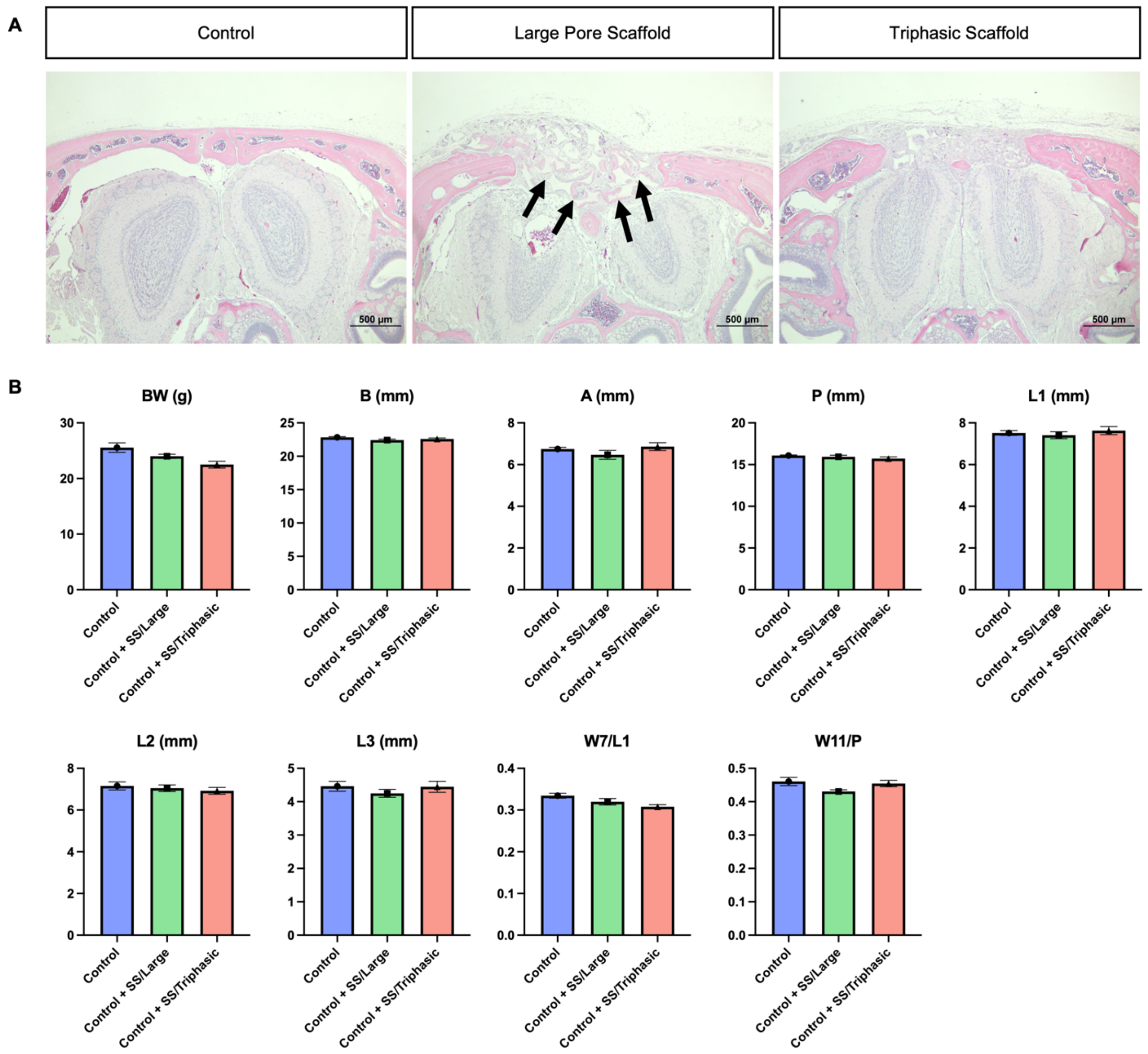

Fig S6 Histologic analysis of control mice to compare suture and engineered suture anatomy of age-matched control. Large scaffold-treated and triphasic scaffold-treated mice at P70 by hematoxylin and eosin stain. Scale bar = 500 um. Black arrows indicate areas of bone formation (**A**). Select morphometric measurements are compared in (**B**). * p<0.05, ** p<0.01, ***p<0.001, ****p<0.0001.

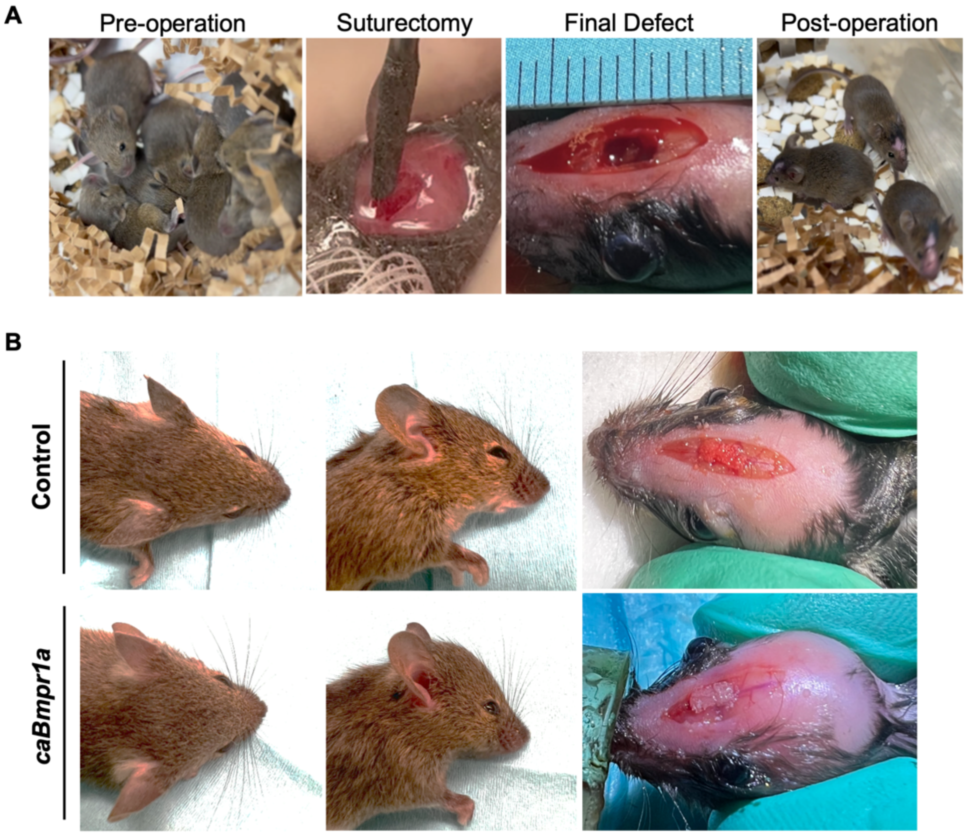

Fig S7 Intraoperative photos of P21 mice treated by SS/TS (**A**) and gross morphology at surgical intervention (**B**).

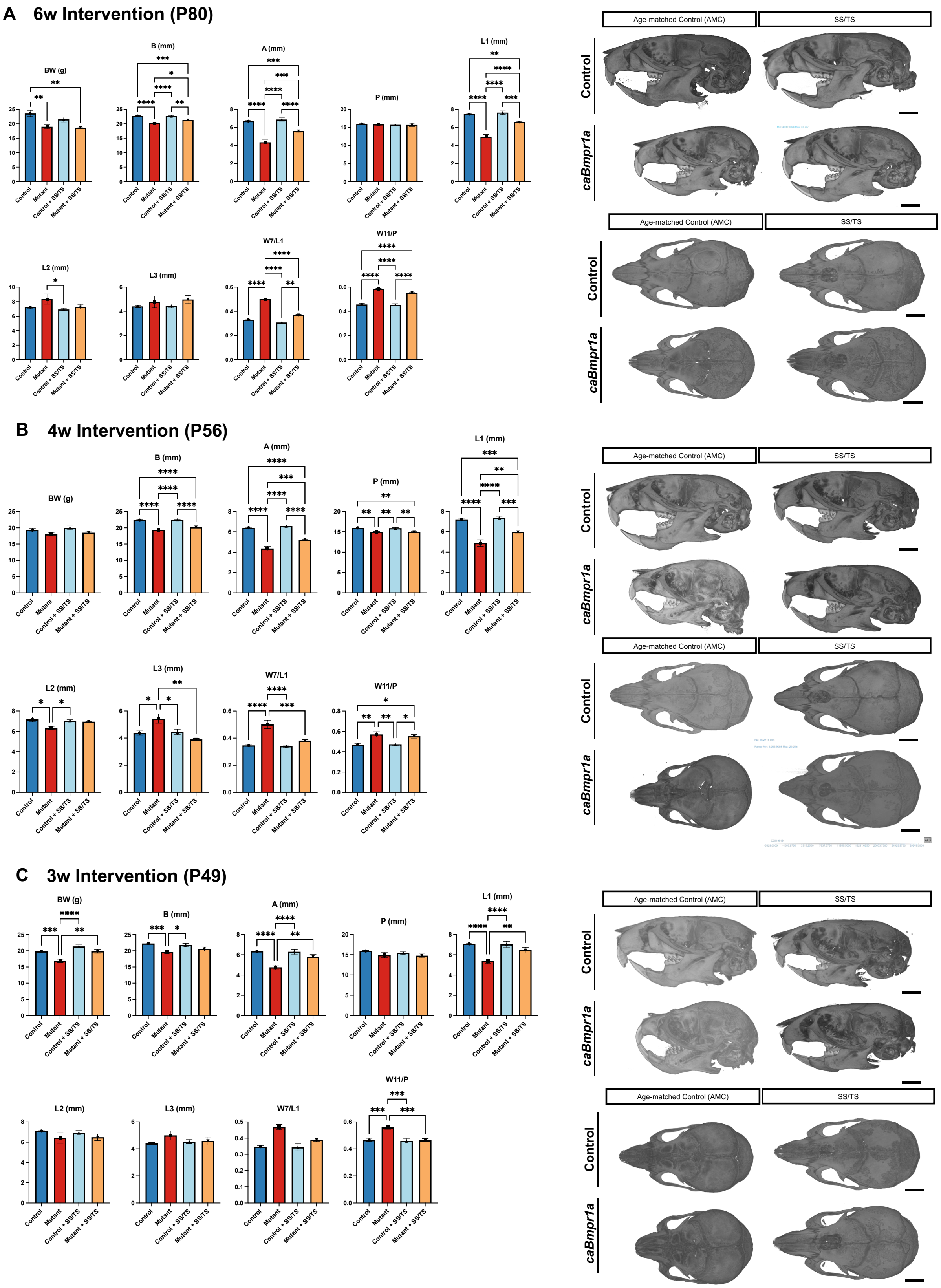
A 6w Intervention (P80)
BW (g)
B (mm)
A (mm)
P (mm)
L1 (mm)
L2 (mm)
L3 (mm)
W7/L1
W11/P
Control
Mutant
Control + SS/TS
Mutant + SS/TS
Age-matched Control (AMC)
SS/TS
caBmpr1a
B 4w Intervention (P56)
C 3w Intervention (P49)

Fig S8 Selected measurements and descriptive statistics describing the craniofacial phenotype of control and caBmpr1a mutant mice, treated with surgical suturectomy and triphasic scaffold implantation compared to non-treated controls, in 6W (**A**), 4W (**B**), and 3W (**C**) intervention groups. * $p<0.05$, ** $p<0.01$, ***$p<0.001$, ****$p<0.0001$. Histologic analysis of control mice was conducted to compare suture and engineered suture anatomy of age-matched, triphasic scaffold-treated mice at P49 (P21 intervention) using hematoxylin and eosin stain (**D**). Scale bar = 500 um.

Table S1 Statistical comparisons of picrosirus red intensities for the four constituent colors at 4 weeks (left) and 8 weeks (right). * p<0.05, ** p<0.01, ***p<0.001, ****p<0.0001.

Table S2 Descriptive statistics comparing control and caBmpr1a mutant mice at P21 (A), P80 (B). * q<0.05, ** q<0.01, ***q<0.001, ****q<0.0001.Principle component analysis comparing untreated control and *caBmpr1a* mutant mice from P21 to P112 (C).

Table S3 Descriptive statistics comparing control and caBmpr1a mutant mice as a function of surgical suturectomy in the P42 intervention cohort. * q<0.05, ** q<0.01, ***q<0.001, ****q<0.0001.

Table S4 Descriptive statistics comparing control and caBmpr1a mutant mice as a function of SS/TS in the P80 intervention cohort. * q<0.05, ** q<0.01, ***q<0.001, ****q<0.0001.

Table S5 Descriptive statistics comparing control mice as a function of SS and large monophasic or triphasic scaffold in the P42 intervention cohort. * q<0.05, ** q<0.01, ***q<0.001, ****q<0.0001.

Table S6 Descriptive statistics comparing control and *caBmpr1a* mutant mice as a function of SS/TS in the P42 (A), P28 (B), and P21 (C) intervention cohort. * q<0.05, ** q<0.01, ***q<0.001, ****q<0.0001.

Table S7 Principle component analysis comparing control and *caBmpr1a* mutant mice, as a function of SS/TS, across all cohorts.

Supplementary Methods:

Scaffold Seeding: Following sterilization and preparation as described above, in a low attachment polystyrene well plate, primary cells (3.0 x 105 cells/scaffold) were seeded onto the 3.5-mm diameter nanofibrous scaffold (3.5 mm diameter 1.0 mm height) used in the bilateral calvarial defect model, or 2.0 x 105 cells/scaffold were seeded onto the 3.5-mm length x 1.5 mm width nanofibrous scaffold used in the suturectomy model, in a volume of 5 uL. Thirty minutes later, culture media was gently added to cover the 3D constructs. Constructs were maintained for 24 hours, and then some (kept as controls) were incubated with culture media containing 10% (v/v) alamarBlue reagent (Invitrogen) for 4 hours. At the end of cultivation, 100 µL of culture media was transferred to a 96-well plate in triplicate for each sample, and levels of 580 nm emission were measured with 530 nm excitation using a fluorescence plate reader to quantify conversion of resazurin to resorufin as a surrogate of attached live cells on the constructs to ensure cell seeding efficiency. Media was changed every 48 hours for scaffolds maintained in vitro. Scaffolds used for in vivo transplantation were transplanted 24 hours after seeding.

In vitro cell migration: Naïve BMSCs were isolated, maintained in growth media (Gibco DMEM) at 37°C, and used in passage 1. Stemness and multipotency were previously demonstrated by Swanson et al., *Biomaterials* 2021 (*36*). To induce osteogenic differentiation, some BMSCs were subjected to 7 days of culture in osteogenic media (DMEM) containing 10% fetal bovine serum, 1% penicillin/streptomycin supplemented with 50 ug/mL ascorbic acid, 10 mM β-glycerophosphate, and 50 ng/mL rh-BMP2 (R&D Systems). Osteogenic differentiation was assessed by Alizarin Red staining in vitro. Naïve and differentiating (BMP-BMSC) BMSCs were trypsinized and cell number determined; Naïve BMSCs were fluorescently labeled with DiO (Vybrant, Thermo Fisher), and BMP-tx BMSCs were fluorescently labeled with DiI (Vybrant, Thermo Fisher) according to the manufacturer's instructions, and washed thoroughly after to remove excess reagent. A total of 2.0 x 10^5 cells were seeded to the center region of the triphasic scaffold; when co-seeded, 1.0 x 10^5 Naïve BMSCs and 1.0 x 10^5 BMP-tx BMSCs were combined first and then seeded at the same time to the center region of the triphasic scaffold, or uniformly in the triphasic scaffold or monophasic large pore scaffold. After 30 minutes, growth media was added to cover the constructs and changed every two days to allow cell adhesion. Cells from male and female donor mice were used; we did not detect differences in male and female cells, and they were combined.

Subcutaneous Implantation Model: Constructs were cultured in media for 24 hours and then implanted subcutaneously into wild-type male mice aged 8-10 weeks. Mice were anesthetized via isoflurane inhalation, and a 2 cm midsagittal incision was made on the dorsal of each mouse. On each side of the midline, two subcutaneous pockets were caused by blunt dissection such that four cell-scaffold constructs were implanted into each mouse in distinct regions. Incisions were closed with sutures, and animals were given analgesic medication (carprofen, 5 mg/kg SC) to manage pain post-operatively for 24 hours. Mice were monitored closely, and no adverse signs were observed. At 4- and 8 weeks following subcutaneous implantation, mice were sacrificed. Constructs were carefully explanted and dissected under a dissecting microscope with an ophthalmic blade, and either fixed in 4% paraformaldehyde before subsequent histologic processing or homogenized in TRIzol (Ambion) for RNA extraction and gene expression analysis.

Gene Expression: Following the manufacturer's instructions, the first strand of cDNA was synthesized from 500 ng of RNA using SuperScript II cDNA Synthesis Kits (Invitrogen). Quantitative polymerase chain reaction (PCR) was performed using Power SYBR Green PCR Master Mix (Applied Biosystems). Gene expression levels were compared among groups with the Applied Biosystems ViiA7 platform. Expression levels of each gene were normalized to endogenous Gapdh. The amplification specificity was confirmed by melting curves.

Lineage Tracing Model: HoxA11Cre^{ERT2}; TdTomato/+; HoxA11eGFP mice were generated by breeding *Hoxa11-Cre^{ERT2}* male mice to *Hoxa11eGFP; ROSATdTomato/TdTomato* female mice. At four weeks of age, mice received 50 mg/kg tamoxifen dissolved in corn oil intraperitoneally, and BMSCs were isolated three days after induction. Femurs and ulna were isolated to confirm transgene expression, and freshly isolated cells were used at passage 1. Ulna sections and BMSCs isolated from mice negative for the mutations were used as a control for establishing background fluorescence signal.

Tamoxifen Induction: At four weeks of age, mice received 50 mg/kg of tamoxifen (Sigma T5648) dissolved in corn oil intraperitoneally. Three days after induction, BMSCs were isolated. Femurs and ulna were also isolated to confirm the expression of transgenes and Cre activities.

HoxA11eGFP BMSC Culture in Scaffolds: As described above, BMSCs were isolated from HoxA11eGFP heterozygous mice, maintained in the growth medium, and used at passage 1 to seed scaffolds. GFP signal was confirmed by histologic section of the ulna and by fluorescence microscopy of the freshly isolated cells. BMSCs isolated from mice negative for the HoxA11eGFP mutation were used as a control for establishing background fluorescence signals in all experiments. To assess the effect of osteogenic differentiation on GFP expression, HoxA11eGFP(+), BMSCs were treated with 50 ng/mL rh-BMP2 in osteogenic media (described above), and the fluorescence signal was measured as a function of time.

Adenoviral-Cre Local Recombination of ALK2Q206D Transgene: Ad5-CMV-cre (Adex-Cre) is obtained from Baylor University (Vector Development Laboratory, Houston, TX) (*49*). 1 x 10^9 PFU Adex-Cre loaded to the scaffold in two 5 uL aliquots, rests 30 minutes at 37°C, then implanted immediately to the mouse subcutaneously or in the bilateral calvarial defect, as described. To avoid cross-cross contamination, all non-Adex-Cre-treated samples are prepared and implanted first, then Adex-Cre-containing samples are prepared and implanted, followed by a thorough decontamination. Mice received all Adex-Cre-containing or all non-Adex-Cre-containing scaffold implants to avoid the potential risk of cross-contamination or leakage.

Calvarial Defect Model: A 3.5-mm bilateral calvarial defect model was used to assess the function of triphasic scaffolds, specifically within the calvarial bone microenvironment. 8-week-old adult mice were anesthetized by isoflurane gas, and the surgical site was prepared for surgery. An 8-10 mm incision along the cranial vault was made, and the periosteum was displaced. Using copious irrigation, a 3.5-mm trephine bur and rotary handpiece were used to create a full-thickness defect in the center of each parietal bone. The defect was placed 1 mm lateral to the sagittal cranial suture. The full-thickness defect was made while respecting the underlying dura mater and brain tissue. Triphasic scaffolds are implanted such that the long axis of the small-pore region is parallel to the long axis of the skull. Scaffolds were stabilized within the defect site, and the overlying tissue was closed with 5-0 Vicryl sutures. Post-operative pain was managed with carprofen (5 mg/kg, SC/IP) for 48 hours. No neurologic defects were observed clinically or histologically. Histologic sections are made perpendicular to the small-pore region's long axis to observe the scaffold's three areas simultaneously.

Histologic Preparation: All samples were either fixed in 4% paraformaldehyde to fix tissue and transferred to 70% ethanol for dehydration before embedding in paraffin (surgical suturectomy model) or fixed in 4% paraformaldehyde to fix tissue and embedded in OCT compound for cryosectioning (calvarial defect models). Mineralized tissues are demineralized with 14% EDTA, pH 7.4, for 14 days at 4°C. Serial sections were cut at 5 um thickness. Standard protocols were followed for hematoxylin and eosin, Masson’s trichrome, and Picrosirius Red staining.

Immunohistochemistry: Deparaffinized sections were incubated in 10 mM citrate buffer (pH 6.0) for antigen retrieval. After treatment with 3% hydrogen peroxide and blocking solution, the sections were incubated with the primary antibody (CD31: Cell Signaling; 1:100 dilution) overnight at 4°C. The sections were treated with HRP-conjugated goat anti-rabbit IgG (Abcam) according to the manufacturer’s instructions. The nuclei were stained with hematoxylin.

Confocal Laser Microscopy: Confocal laser microscopy uses a Nikon Eclipse T2 instrument.

Microcomputed Tomography (micro-CT): For micro-CT analysis, dissected samples were placed in a 19-mm-diameter specimen holder and scanned over the entire length using a micro-CT system (μCT100 Scanco Medical, Bassersdorf, Switzerland). The scan settings were voxel size 12 μm, 70 kVp, 114 μA, 0.5 mm AL filter, and integration time 500 ms. Files were exported as ISQ and DICOM for subsequent analysis. Analysis was carried out using Scanco Medical software for reconstruction, evaluation, and 3D analysis (Scanco).
